# Netgram: Visualizing Communities in Evolving Networks

**DOI:** 10.1371/journal.pone.0137502

**Published:** 2015-09-10

**Authors:** Raghvendra Mall, Rocco Langone, Johan A. K. Suykens

**Affiliations:** KU Leuven, ESAT-STADIUS, Kasteelpark Arenberg 10, B-3001 Leuven, Belgium; University of Namur, BELGIUM

## Abstract

Real-world complex networks are dynamic in nature and change over time. The change is usually observed in the interactions within the network over time. Complex networks exhibit community like structures. A key feature of the dynamics of complex networks is the evolution of communities over time. Several methods have been proposed to detect and track the evolution of these groups over time. However, there is no generic tool which visualizes all the aspects of group evolution in dynamic networks including birth, death, splitting, merging, expansion, shrinkage and continuation of groups. In this paper, we propose Netgram: a tool for visualizing evolution of communities in time-evolving graphs. Netgram maintains evolution of communities over 2 consecutive time-stamps in tables which are used to create a query database using the sql outer-join operation. It uses a line-based visualization technique which adheres to certain design principles and aesthetic guidelines. Netgram uses a greedy solution to order the initial community information provided by the evolutionary clustering technique such that we have fewer line cross-overs in the visualization. This makes it easier to track the progress of individual communities in time evolving graphs. Netgram is a generic toolkit which can be used with any evolutionary community detection algorithm as illustrated in our experiments. We use Netgram for visualization of topic evolution in the NIPS conference over a period of 11 years and observe the emergence and merging of several disciplines in the field of information processing systems.

## Introduction

Large scale complex networks are ubiquitous in the modern era. Their presence spans a wide range of domains including social networks [1], biological networks [2], collaboration networks [3], trust networks [4] and communication networks [5]. These complex networks have a natural temporal aspect. Social networks evolve over time with addition and deletion of members, formation of friendships between people in different social circles or disappearance of friendship between people over time. In collaboration networks, a group of researchers working on a particular topic might collaborate intensively if they are working on an emerging topic whereas a group of researchers working together on an outdated topic might completely disappear over time.

Complex networks can be represented as graphs *G* = (*N*, *E*) where *N* represent the vertices or nodes and *E* represents the edges or interaction between these nodes in the network. Most real-life networks exhibit community like structure i.e. nodes within a community are more densely connected to each other and sparsely connected to nodes outside that cluster. Traditionally, community detection methods [5–17] have focused on identifying communities in static representations of graphs.

However, by representing a time-evolving graph as a static network [10] it becomes difficult to detect and track the intrinsic changes in the community structures over time. By performing community detection on static snapshots [10] of the dynamic network, we loose the property of temporal smoothness which is essential to capture the evolution of communities over time. Temporal smoothness [18–20] allows to preserve the long-term trend in the dynamic graph while smoothing out short-term variations due to noise. This property is similar to the property of moving averages in time-series analysis [20]. In recent years, the problem of evolutionary community detection and its tracking in large scale dynamic social networks has received a lot of attention [19–29]. The goal of evolutionary community detection is to identify and track communities at different snapshots of time in non-stationary graphs. Throughout this paper we use the notation T to denote total number of time-stamps and *t* to denote an arbitrary time-stamp *t* ≤ T. We use $C_{j}^{t}$ which comprises a set of nodes to represent the *j* ^*th*^ cluster at time-stamp *t* and 𝓒^*t*^ (which is union of all $C_{j}^{t}$) to represent the set consisting of all the clusters $C_{j}^{t}$ at time-stamp *t*.

**Fig 1:**
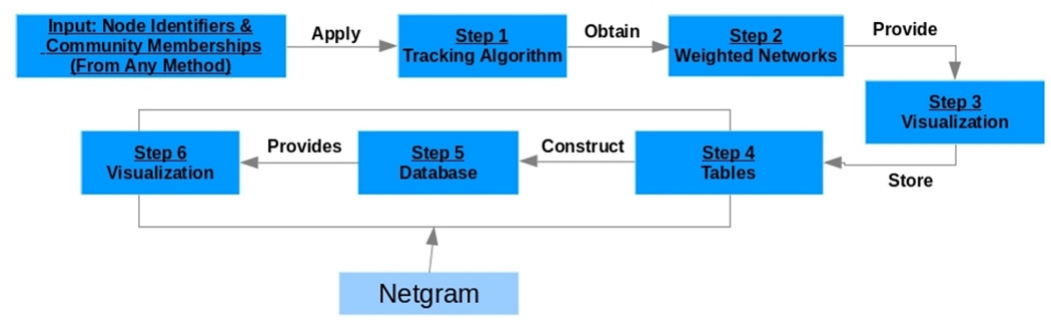
Steps undertaken by Netgram for visualizing evolution of communities in dynamic networks.

**Fig 2:**
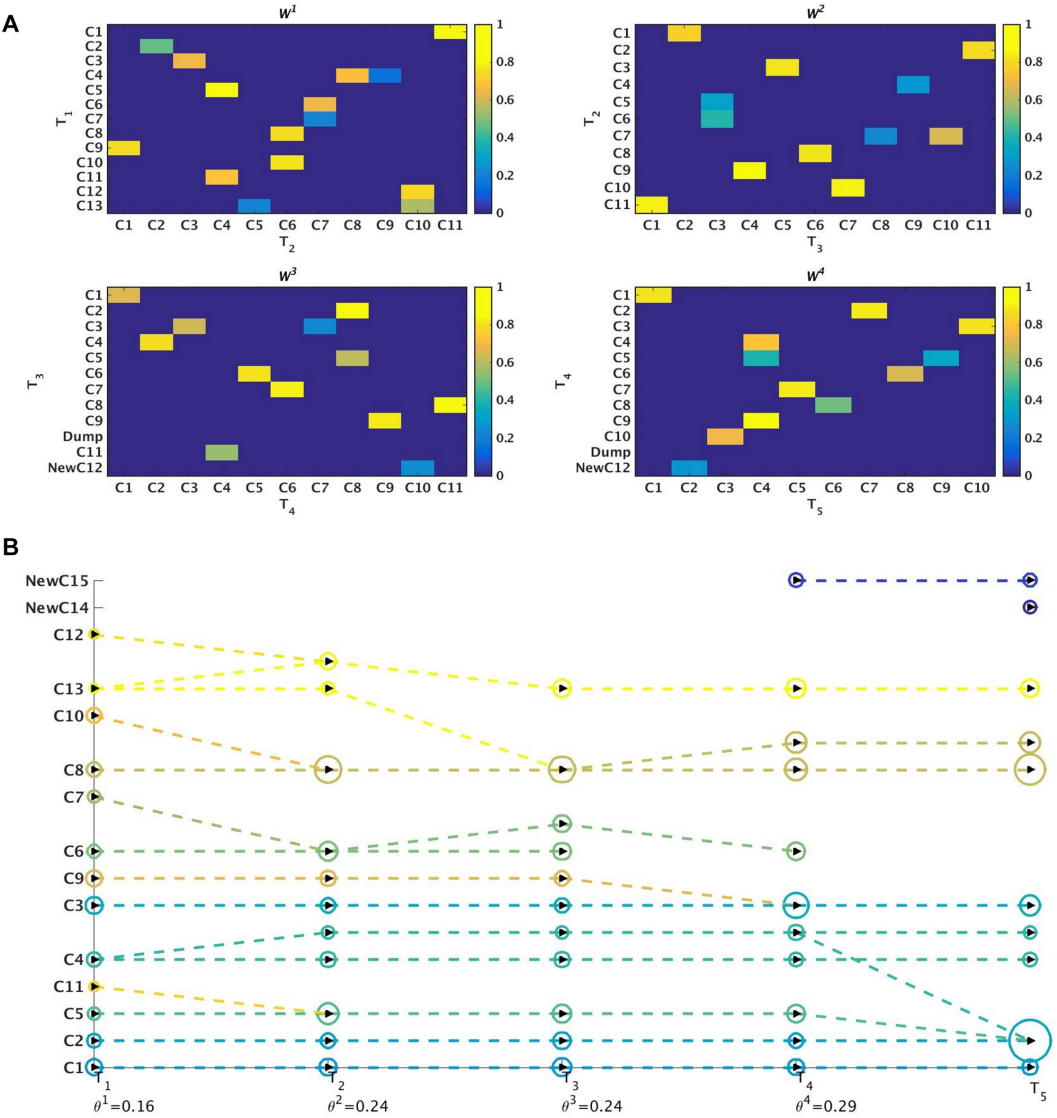
a) Visualization of the weighted networks (*W* ^*t*^) mapping evolution of communities over 5 time-stamps. *W* ^*t*^ tracks evolution of a cluster between two consecutive time-stamps. The colors represent the weight of the edges in *W* ^*t*^. The weights can take value in the range [0, 1] i.e. 0 ≤ *w*(V^*t*^(*j*, *k*)) ≤ 1. b) Visualization and tracking of community evolution by Netgram for the clusters obtained from the Kernel Spectral Clustering with Memory Effect (MKSC) algorithm [28] for Birthdeath dataset. Netgram showcases the birth, death, merge, split, expansion, shrinkage and continuation of communities for the Birthdeath dataset over 5 time-stamps (T_1_, T_2_, T_3_, T_4_ and T_5_). We represent each community with a different colour circle and the size is ∝ the number of nodes in that community. From Fig 2b, we can observe the death of clusters **C6** & **C7** at time-stamps T_3_ and T_4_ respectively. Similarly, we can see birth of clusters **NewC14** & **NewC15** at time-stamp T_4_ and T_5_ respectively. We also observe that cluster **C11** merges with **C5** at time-stamp T_2_ and cluster **C8** splits into 2 clusters at time-stamp T_4_. We can observe that cluster **C8** has expanded at time-stamp T_3_. The cluster **C8** also contracts at time-stamp T_4_ as it splits into 2 clusters. Cluster **C1** demonstrates continuation over time.

In this paper, we propose a new tool Netgram which allows to visualize the evolution of communities in dynamic graphs. In order to visualize the progress of communities over time, Netgram follows a series of steps. Netgram takes as input the information about the individual identifier for all the nodes i.e. the number/label with which that node is represented in the network and its corresponding cluster membership at different time-stamps. It can be used as a post-processing step to any evolutionary community detection algorithm. After obtaining the input, in order to capture the significant events namely birth, death, merge, split, growth, shrinkage and continuation of communities, Netgram uses a modified version of the tracking algorithm proposed in [29]. We provide a visualization of the weighted network (*W* ^*t*^) at time-stamp *t* generated as a result of the tracking procedure. Once the evolution of communities is captured between two successive time-stamps, it is stored in a table. We then perform sql [30] join operations on these sets of tables to construct a query database. This query database contains information about evolution of all the communities over the different time-stamps. We then visualize this query database using separate colors for cluster identifiers and lines which track the evolution of individual communities. Netgram tries to adhere to certain design principles and a set aesthetic guidelines including minimizing the line cross-overs between the evolving communities during events like merge and split making it easier to track the evolution of individual communities in the dynamic network. The problem of minimizing the cross-talk between communities during different time-stamps is combinatorial by nature and Netgram uses a greedy solution for the same. Fig 1 illustrates the steps undertaken by the Netgram toolkit for visualizing evolution of communities. Fig 2 showcases the result that we get from the Netgram toolkit on a synthetic Birthdeath dataset of 1,000 nodes over 5 time-stamps generated from the software https://github.com/derekgreene/dynamic-community.

## Related Work

We briefly mention here some of the methods that have been used in the past for detecting and tracking changes in complex networks. In [27] GraphScope was introduced. Graphscope is an efficient adaptive mining tool in time evolving graph for detecting communities. It requires no user-defined parameter and operates completely in a standalone mode using the principle of Minimum Description Length (MDL) from information theory. Moreover, it can automatically detect communities and determine good *change-points* over time. The intuition is that communities do not change much over 2 consecutive time-stamps and thus have similar description lengths. This allows to group them together into a time segment in order to achieve better compression. Whenever a new snapshot cannot fit well into the old segment (in terms of compression), it introduces a change-point and starts a new segment at that time-stamp. These change-points detect drastic discontinuities in time.

In [25], the authors provided a framework called FaceNet. In this technique, the authors deviated from the traditional two-step approach to analyze community evolutions. In the traditional approach, communities are first detected for each time-stamp and then compared to determine correspondences. In this approach, the authors used a framework called FaceNet for analyzing communities and their evolutions through a robust unified process. This framework allowed the discovery of communities and captured their evolution with temporal smoothness [19] given by the historic community structures. The authors formulated their problem in terms of maximum a posteriori (MAP) estimation, where the community structure was estimated both by the observed networked data and by the prior distribution given by historic community structures. Then an iterative algorithm was developed which guaranteed convergence to an optimal solution.

In both the aforementioned techniques namely Graphscope [27] and FaceNet [25], the primary focus was on detection of communities in time evolving graphs rather than tracking the evolution of communities. These techniques can identify significant events like birth, death and continuation of communities. However, it is difficult to detect significant events like merge and split using these techniques.

In [24], a framework was provided which used the clique percolation method (CPM) for tracking evolution of communities in successive time-stamps. However, this technique is prone to be affected by noisy events. For example, if very few nodes from a community at one time-stamp split then this method detects it as a split event whereas these nodes might have been removed due to random fluctuations in community detection technique. In [22], a model was introduced which tracked the progress of communities over time in a dynamic network such that each community is characterized by a series of significant evolutionary events. By only keeping track of significant evolutionary events they overcome noisy events. However, none of these methods [22, 24] provide a visualization tool to observe and get insight into the evolution of communities in dynamic networks.

Recently, a method which maps changes in networks using alluvial diagrams was proposed in [21]. The method uses bootstrap sampling accompanied by significance clustering in order to distinguish meaningful structural changes from random fluctuations. This technique is based on the principle introduced in [22]. However, this method [21] doesn’t take into consideration the property of temporal smoothness [19]. The alluvial diagrams help to visualize events like merge, split, continuation, expansion and shrinkage of communities. However, they cannot showcase the birth and death of individual communities separately. This is because in this method [21] a new community can only emerge at a given time-stamp *t* as a split of some previous community at time-stamp *t* − 1 and it is difficult to detect the death/dissolution of a particular community at one given time-stamp *t*. Moreover, this visualization using surfaces uses a large portion of the screen and makes it difficult to track the evolution of individual communities when there is a series of merge and split events. Netgram allows to separately identify birth of a new community, highlight the death of a community and makes it easy to identify and track individual community evolution in presence of multiple merge and split events using a simple line-based visualization system. The tracking algorithm of Netgram is based on similar principles as that proposed in [21, 22, 29] i.e. trying to identify significant events and differentiate it from random noisy events. However, the main goal of Netgram is to come up with a simple line-based visualization tool which allows to track evolution of individual communities, capture and visualize significant events like birth, death, merge, split, continuation, shrinkage and growth of communities in dynamic time-evolving graphs.

## Evolution of Communities

Significant events that happen during evolution of communities in a dynamic network can be defined as:

- *Birth*: The emergence of a new community $C_{new}^{t}$ at time *t* comprising nodes which were previously unseen at time *t* − 1 i.e. $C_{new}^{t}\cap C_{i}^{t-1}=\emptyset$ with all the clusters $C_{i}^{t-1}$ in the set 𝓒^*t*−1^ at time *t* − 1.
- *Death*: The disappearance of one or more communities at time *t*. It suggests that the community which was appearing as $C_{i}^{t-1}$ at a previous time has disappeared now i.e. $\frac{|C_{i}^{t-1}\cap C_{j}^{t}|}{|C_{i}^{t-1}\cup C_{j}^{t}|}<\theta^{t-1}$ for all the communities $C_{j}^{t}$ in the set 𝓒^*t*^ at time *t*.
- *Continuation*: A community $C_{i}^{t-1}$ remains intact for the next time *t* i.e $\frac{|C_{i}^{t-1}\cap C_{j}^{t}|}{|C_{i}^{t-1}\cup C_{j}^{t}|}\geq \theta^{t-1}$ holds true for a single community $C_{j}^{t}$ at time *t* and it holds true in both time-directions. This distinguishes a continuation event from a merge event. Since, the community structure of $C_{i}^{t-1}$ doesn’t change much and continues to remain a single community $C_{j}^{t}$ at time *t*, it is referred as a continuation.
- *Merging*: When the majority of the nodes from 2 or more communities at time *t* − 1 for example $C_{h}^{t-1}$ and $C_{i}^{t-1}$ combine together to form one cluster $C_{j}^{t}$ at time *t*. i.e. $\frac{|C_{h}^{t-1}\cap C_{j}^{t}|}{|C_{h}^{t-1}\cup C_{j}^{t}|}\geq \theta^{t-1}$ and $\frac{|C_{i}^{t-1}\cap C_{j}^{t}|}{|C_{i}^{t-1}\cup C_{j}^{t}|}\geq \theta^{t-1}$, all such conditions hold true. These clusters subsequently start to share a common time-line starting from time *t*.
- *Splitting*: When the majority of the nodes from a community $C_{i}^{t-1}$ splits into 2 or more communities at time *t* say $C_{j}^{t}$ and $C_{k}^{t}$ i.e. $\frac{|C_{i}^{t-1}\cap C_{j}^{t}|}{|C_{i}^{t-1}\cup C_{j}^{t}|}\geq \theta^{t-1}$ and $\frac{|C_{i}^{t-1}\cap C_{k}^{t}|}{|C_{i}^{t-1}\cup C_{k}^{t}|}\geq \theta^{t-1}$, all such conditions hold true.
- *Growth*: When the number of nodes in the community $C_{i}^{t-1}$ at time *t* − 1 increase significantly for the corresponding community $C_{j}^{t}$ at time *t*. For example, if the size of the community $C_{i}^{t-1}$ increases by 20% at time *t*.
- *Shrinkage*: When the number of nodes in the community $C_{i}^{t-1}$ at the time *t* − 1 decreases significantly for the corresponding community $C_{j}^{t}$ at time *t*. For example, if the size of the community $C_{i}^{t-1}$ decreases by 20% at time *t*.
  The parameter *θ* ^*t* − 1^ is defined for each time-stamp *t* ≤ T. It helps to define the concept of majority or helps to keep track of events which are significant and prevents random fluctuations from being detected as merge or split events. It takes value between the interval [0, 1] and is explained in more detail in the next subsection.

### Tracking Algorithm & Weighted Networks

In order to recognize these events we need a tracking algorithm that matches the communities found by the evolutionary clustering algorithms at each time step. Several such tracking algorithms have been developed including [22, 24, 29]. In this paper, we use a modified version of the tracking algorithm introduced in [29].

We first generate a weighted directed bipartite network *W* ^*t*^ from the clusters at two consecutive time-stamps *t* and *t* + 1. In case of a total of T time-stamps, we generate a set 𝓦 = {*W* ^1^, …, *W* ^T−1^} of weighted directed bipartite networks. Each bipartite network *W* ^*t*^ creates a map between the set of clusters at time-stamp *t* i.e. 𝓒^*t*^ and time-stamp *t* + 1 i.e. 𝓒^*t*+1^. Here ${𝓒}^{t}=\{C_{1}^{t},\ldots ,C_{n}^{t}\}$, where *n* represents total number of clusters at time-stamp *t*. This map corresponds to the edges of the network. The weight *w*(V^*t*^(*j*, *k*)) of an edge V^*t*^(*j*, *k*) between two clusters $C_{j}^{t}$ and $C_{k}^{t+1}$ corresponds to the fraction of nodes in cluster *C*_*j*_ at time-stamp *t* and cluster *C*_*k*_ at time-stamp *t* + 1 which are assigned to cluster *C*_*k*_ at time-stamp *t* + 1 and is represented as:

$$\begin{array}{c} w\left({\text{V}}^{t}\left(j,k\right)\right)=\frac{|C_{j}^{t}\cap C_{k}^{t+1}|}{|C_{j}^{t}\cup C_{k}^{t+1}|} \end{array}$$ (1)

where the numerator is equal to the number of nodes of cluster $C_{j}^{t}$ which are also part of $C_{k}^{t+1}$ and $|C_{j}^{t}\cup C_{k}^{t+1}|$ represents the total number of distinct nodes in clusters $C_{j}^{t}$ and $C_{k}^{t+1}$. This fraction is equivalent to the widely-adopted Jaccard co-efficient [31]. This edge-weighting scheme is similar to the one proposed in [22] and was shown to successfully capture significant events. This edge weight calculation scheme is another method to track evolution of communities between 2 time-stamps and is different from the one proposed in [29]. It gives importance to both the nodes in $C_{j}^{t}$ at time-stamp *t* and the nodes in $C_{k}^{t+1}$ at time-stamp *t* + 1.

An edge exists between two clusters $C_{j}^{t}$ and $C_{k}^{t+1}$ only if *w*(V^*t*^(*j*, *k*)) > 0. We then create an empty list *L* ^*t*^. Here we keep the information about the maximum weighted outgoing edge from each cluster $C_{i}^{t}$ at time-stamp *t* i.e. argmax_*j*_ *w*(V^*t*^(*i*, *j*)) and the maximum weighted incoming edge for each cluster $C_{j}^{t+1}$ at time-stamp *t* + 1 i.e. argmax_*i*_ *w*(V^*t*^(*i*, *j*)). The list *L* becomes:

$$\begin{array}{c} L^{t}=\left[w\left({\text{V}}^{t}\left(i,{\text{argmax}}_{j}w\left({\text{V}}^{t}\left(i,j\right)\right)\right)\right),\ldots ,w\left({\text{V}}^{t}\left({\text{argmax}}_{i}w\left({\text{V}}^{t}\left(i,j\right)\right),j\right)\right),\ldots \right], \end{array}$$

where *i* = 1, …, ∣𝓒^*t*^∣ and *j* = 1, …, ∣𝓒^*t*+1^∣. Here *i* and *j* vary from 1 to the total number of clusters in the set 𝓒^*t*^ and 𝓒^*t*+1^ respectively. List *L* ^*t*^ has all the maximum weighted edges from all $C_{i}^{t}\subset {𝓒}^{t}$ and all the maximum weighted edges to *C*_*j*_ ⊂ 𝓒^*t*+1^. We then define a threshold $\theta^{t}=L_{k}^{t}$ where $k={\text{argmin}}_{l}L_{l}^{t}$, *l* = 1, …, ∣*L*∣ i.e. it is the minimum weight in the list *L* ^*t*^ of maximum weighted edges in the bipartite graph *W* ^*t*^. We use this minimum weight *θ* ^*t*^ to define the concept of majority or only those edges are kept in *W* ^*t*^ whose edge weights are greater than or equal to *θ* ^*t*^. By selecting the minimum of all the maximally weighted edges (i.e. in-coming as well as out-going edges) between all the communities for 2 consecutive time-stamps, we even allow communities of smaller densities to have in-coming or out-going edges. If instead we take the average of all the maximally weighted edges for all the communities in these 2 time-stamps, we run into the risk of missing edges to and from communities of smaller density to communities of larger density. The average edge-weight criterion is influenced more by outliers or extreme edge-weights values. Thus, this criterion based on selecting the minimum of all the maximally weighted edges, acts an efficient tool a) prevent random noisy fluctuations from being detected as split or merge events and b) to detect various significant events for communities of different densities.

If the number of edges going out of a node of *W* ^*t*^ is greater than 1, it indicates the possibility of a split at time *t* + 1, whereas if the number of edges entering a node of *W* ^*t*^ at time *t* + 1 is greater than 1 then it indicates the possibility of a merge. A split will only happen if the corresponding clusters say $C_{k}^{t+1}$ and $C_{l}^{t+1}$ have an edge weight *w*(V^*t*^(*j*, *k*)) ≥ *θ* ^*t*^ and *w*(V^*t*^(*j*, *l*)) ≥ *θ* ^*t*^ respectively coming from cluster $C_{j}^{t}$. Similarly, a merge will only happen if weight of the edges from clusters say $C_{i}^{t}$ and $C_{j}^{t}$ to cluster $C_{k}^{t+1}$ are greater than *θ* ^*t*^.

We observe continuation of a community when majority of the nodes from a cluster $C_{j}^{t}$ are part of the some cluster at time *t* + 1 say cluster $C_{k}^{t+1}$ i.e. *w*(V^*t*^(*j*, *k*)) ≥ *θ* ^*t*^. In order to tackle the death of one or more communities, we add a *Dump* cluster to the set of clusters 𝓒^*t*^ for each time-stamp *t* (except time 1 when we are first identifying the communities). The *Dump* cluster represents death of a community. Similarly, in order to handle birth of a new cluster from nodes which were previously unseen at time *t*, we add a cluster $C_{0}^{t}$ to the set of clusters 𝓒^*t*^ for each time-stamp *t* except the first time-stamp. If more than one cluster is born at a given time-stamp *t* then we use identifiers $C_{0}^{t}$, $C_{-1}^{t}$, $C_{-2}^{t}$ etc. for the newborn clusters. This allows to overcome the problem of detection of events like birth and death faced by the tracking algorithm in [29]. In [29], the authors used the same identifier for birth and death of communities which can lead to confusion when there is birth and death of a community at a given time-stamp. Moreover, this technique [29] cannot identify birth of multiple communities at a given time-stamp.

**Fig 3:**
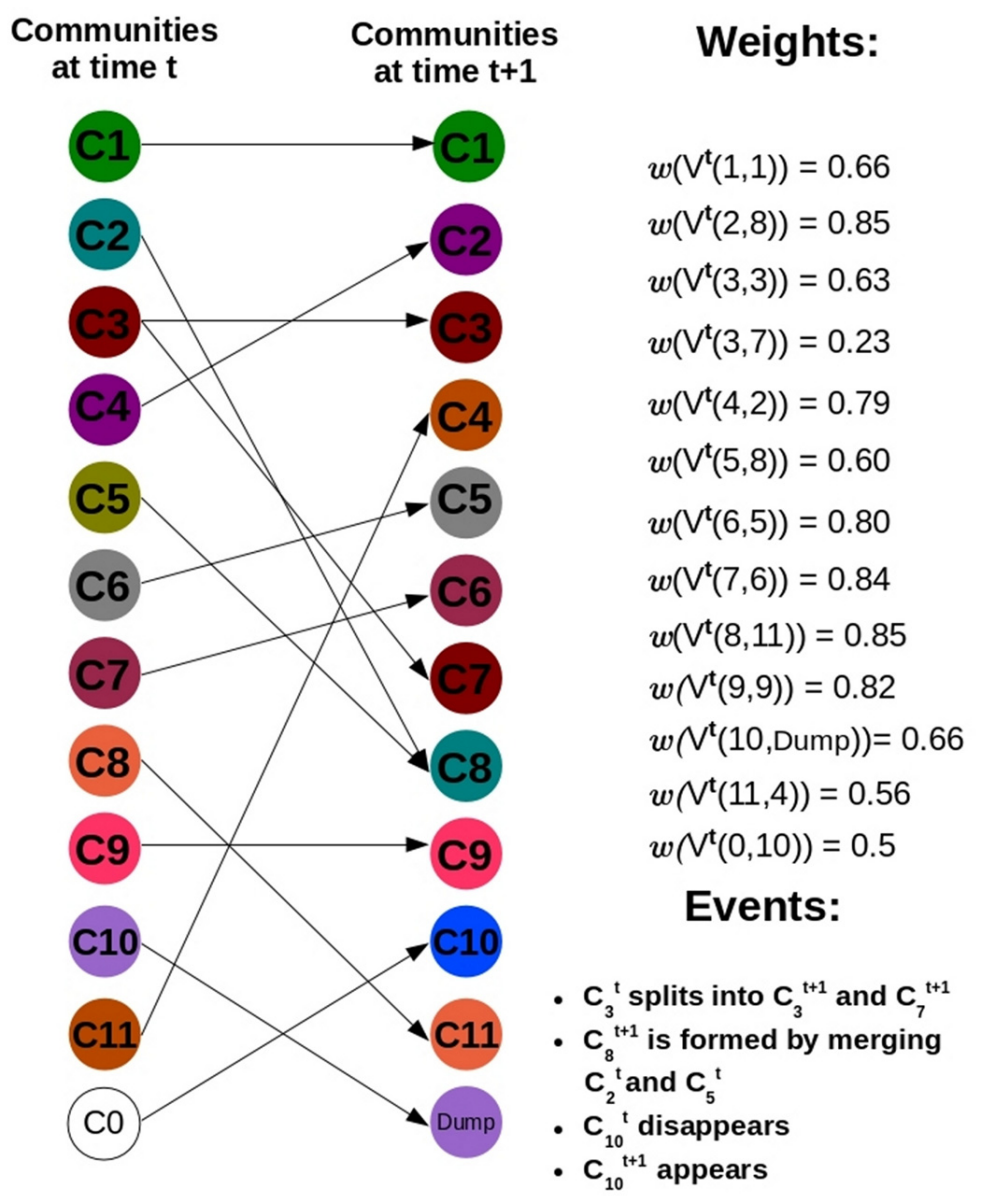
Weighted directed bipartite network *W* ^*t*^ corresponding to 2 consecutive time-stamps for the synthetic Birthdeath dataset.

**Fig 4:**
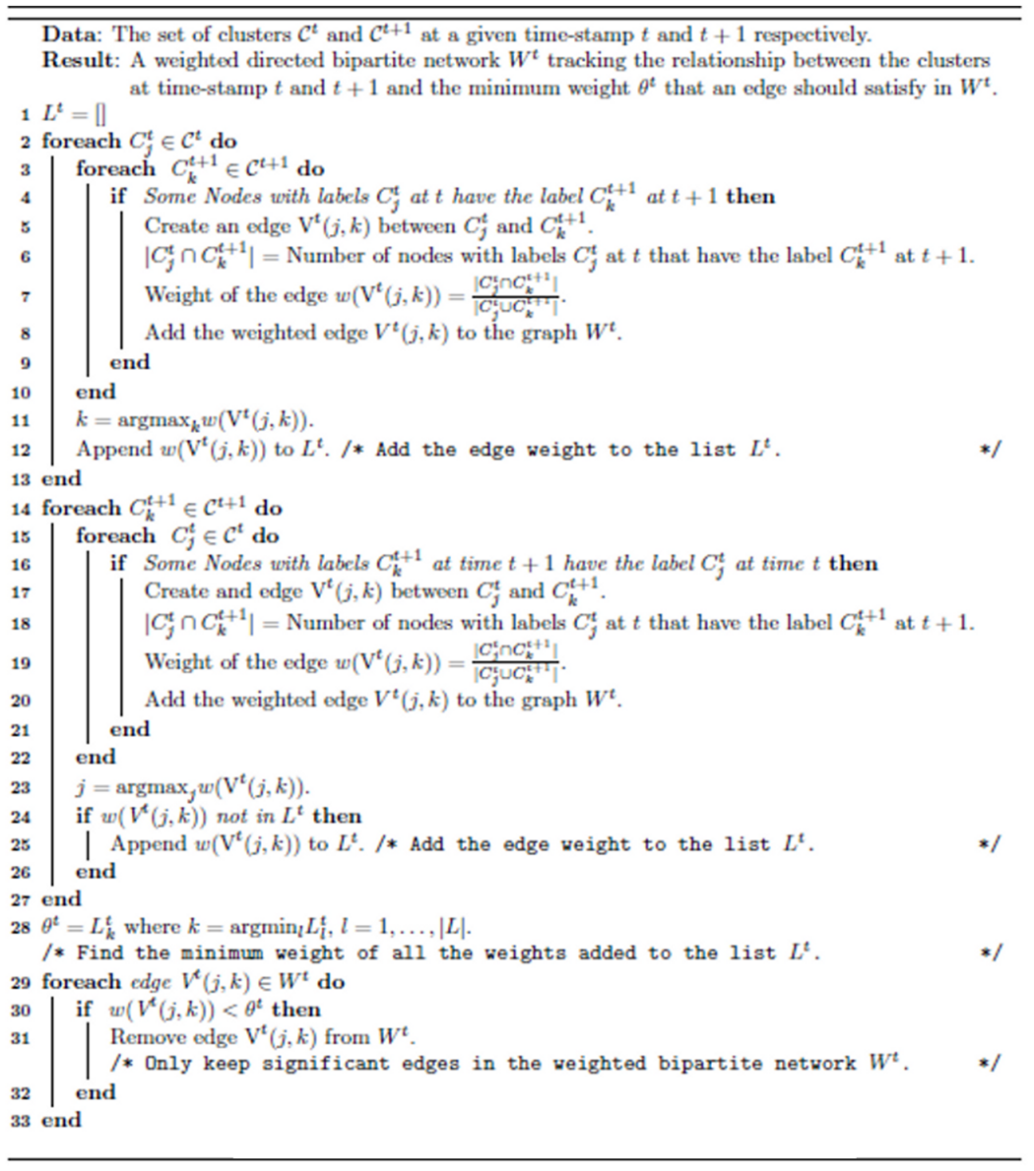
Algorithm 1: Community Tracking Algorithm.

For the network *W* ^*t*^, if $C_{0}^{t}$ is isolated then no new clusters were generated at time *t* and if $C_{0}^{t}$ has an outgoing edge with weight ≥ *θ* ^*t*^ then a new community has emerged at time *t* + 1. Similarly, if we have incoming edges to *Dump* at time *t* + 1 in *W* ^*t*^ then some clusters have disappeared. An advantage of having separate identifiers for tracking the birth and the death of communities is that it becomes easier to distinguish the two events when we are performing sql join operations [30] to generate the query database. Fig 3 gives an example of the mapping mechanism for 2 snapshots of Birthdeath dataset. This tracking procedure is summarized in Algorithm 1 as shown in Fig 4.

### Visualizing Weighted Bipartite Networks

After obtaining the set 𝓦, we visualize the weighted bipartite networks *W* ^*t*^ for each time-stamp *t*. The steps involved in the visualization include using each bipartite network *W* ^*t*^ as an adjacency matrix *A* ^*t*^ and plotting the adjacency matrix as an image.

An edge in the adjacency matrix *A* ^*t*^(*i*, *j*) indicates the connection between cluster $C_{i}^{t}$ at time *t* and cluster $C_{j}^{t+1}$ at time *t* + 1 and the weight of the edge is *A* ^*t*^(*i*, *j*) = *w*(V^*t*^(*i*, *j*)) obtained from *W* ^*t*^. For the purpose of visualization we remove all the edges directed to the *Dump* cluster. Let us say that cluster $C_{i}^{t}$ disappeared at time *t* + 1. Then, sum of the weight of all the edges from community $C_{i}^{t}$ to all the clusters at time-stamp *t* + 1 is 0. Hence, it becomes easier to identify communities which have disappeared. In the plot of *A* ^*t*^, we replace cluster $C_{i}^{t}$ with *Dump*. In case of birth of one or more new communities we look up the entries corresponding to *A* ^*t*^(0, *i*), *A* ^*t*^(−1, *j*) etc. in the adjacency matrix at time-stamp *t* + 1. In the plot of *A* ^*t*^, we indicate these newborn clusters with the prefix ‘NewC’.

**Fig 5:**
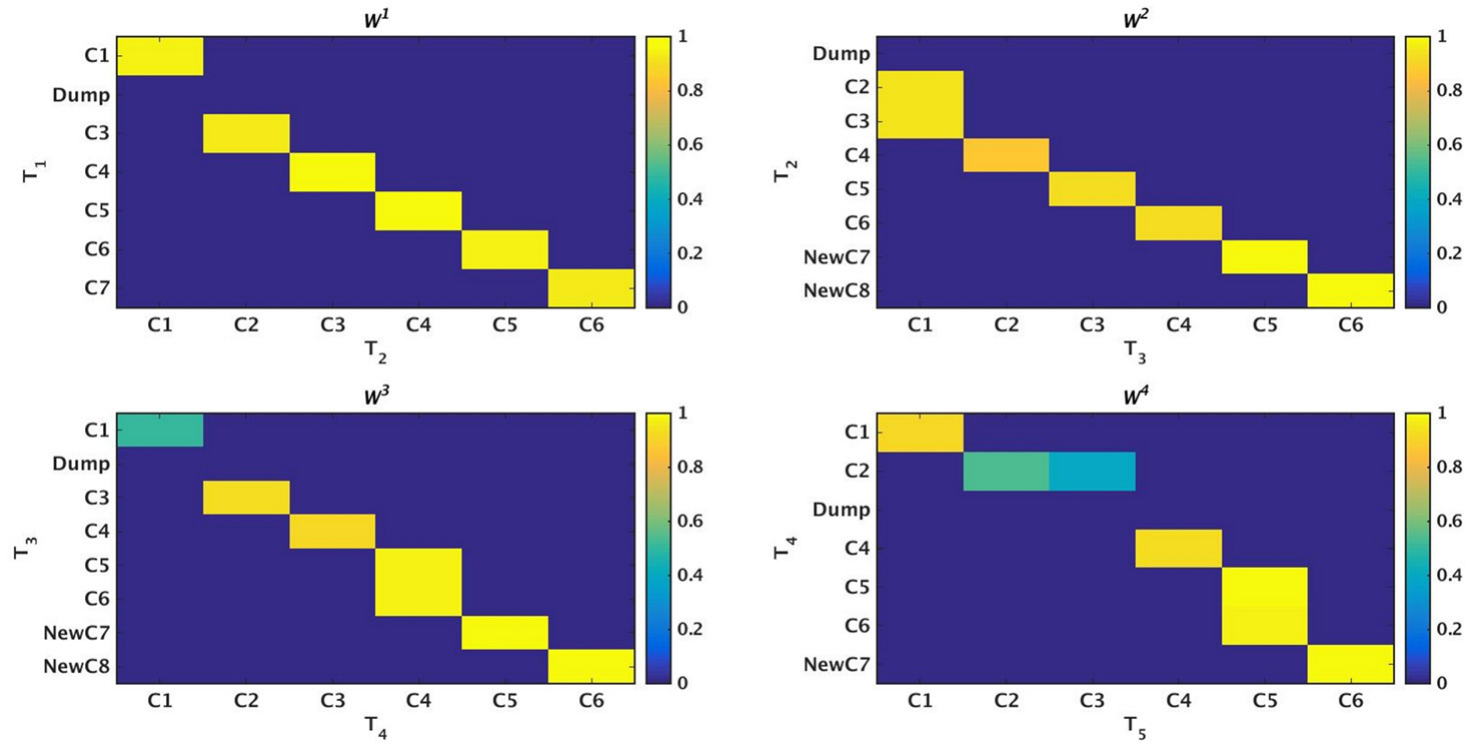
Visualization of the weighted networks *W* ^*t*^ mapping the evolution of communities over 5 time-stamps. *W* ^*t*^ tracks the evolution of a cluster between two successive time-stamps. We can observe that at each time-stamp T_*i*_ there is death of one community. We can also detect the birth of one or more communities at each time-stamp T_*i*_, *i* > 2. The *x*-axis and *y*-axis represent the set of clusters at two consecutive time-stamps. The colors represent the weight of the edges for *W* ^*t*^.

An example of this visualization procedure is shown in Fig 5 in the case of a synthetic Hide dataset. This Hide dataset comprises 5 snapshots where one community disappears at each time-stamp *t* and one or more communities appear at each time-stamp *t* > 1. We use the evolutionary community detection algorithm introduced in [23] (namely Evolutionary Spectral Clustering) to illustrate that the proposed visualization also works for another evolutionary clustering algorithm.

## Netgram Tool

Once we have obtained the set 𝓦, we store the connections between nodes of the bipartite network *W* ^*t*^ at time *t* in tabular format. For the sake of simplicity, we just keep the information about the source and the sink of the edge i.e. for an edge *V* ^*t*^(*j*, *k*), we keep (*j*, *k*) in table *P* ^*t*^ for time *t*. This results in a set of tables 𝓟 = {*P* ^1^, …, *P* ^T−1^} for T time-stamps.

We then construct a query database 𝓓 by performing an outer-join sql operation [30, 32] between table *P* ^1^ and *P* ^2^ using the unique identifiers in 2^*nd*^ column of *P* ^1^ and 1^*st*^ column of *P* ^2^ as keys. We then repeat the process with query database 𝓓 and succeeding tables in set 𝓟 i.e. *P* ^3^, …, *P* ^T−1^ using the unique identifiers of last column of database 𝓓 and 1^*st*^ column of table *P* ^*i*^ (*i* > 2) as keys on which the outer-join operation [30] is performed. By keeping separate unique identifiers for a dead cluster and a newborn community, it becomes easier to track birth and death of communities over time.

An example of the query database 𝓓 obtained as a result of this process is shown in Table 1 for a synthetic Mergesplit dataset using the OSLOM [11] method. Since the OSLOM method is a hierarchical community detection algorithm, we only track large size communities at coarser levels of hierarchy for the 5 snapshots of the Mergesplit dataset.

**Table 1:** Tracking the 7 communities detected by the OSLOM method [11] at time-stamp T1 for Mergesplit dataset. This tracking information is stored in database 𝓓 which is depicted here.

T1	T2	T3	T4	T5
*C*_1_	*C*_7_	*C*_3_	*C*_1_	*C*_3_
*C*_2_	*C*_1_	*C*_5_	*C*_7_	*C*_5_
*C*_2_	*C*_2_	*C*_5_	*C*_7_	*C*_5_
*C*_3_	*C*_4_	*C*_2_	*C*_2_	*C*_1_
*C*_3_	*C*_4_	*C*_2_	*C*_3_	*C*_1_
*C*_3_	*C*_3_	*C*_8_	*C*_6_	*C*_2_
*C*_3_	*C*_3_	*C*_1_	*C*_9_	*C*_7_
*C*_3_	*C*_3_	*C*_1_	*C*_9_	*C*_8_
*C*_4_	*C*_6_	*C*_9_	*C*_4_	*C*_6_
*C*_4_	*C*_6_	*C*_9_	*C*_10_	*C*_4_
*C*_4_	*C*_6_	*C*_9_	*C*_10_	*C*_11_
*C*_5_	*C*_8_	*C*_6_	*C*_1_	*C*_3_
*C*_6_	*C*_8_	*C*_6_	*C*_1_	*C*_3_
*C*_7_	*C*_5_	*C*_4_	*C*_5_	*C*_9_
*C*_7_	*C*_5_	*C*_7_	*C*_8_	*C*_10_

We use a simple line-based tracking mechanism to visualize the query database 𝓓. This line-based tracking is inspired by dendograms [33] which are used for visualization of layers of hierarchy in hierarchical clustering algorithms [15, 34, 35]. Similarly, the concept can be applied in case of dynamic networks where the layers represent the individual time-stamps for the evolving network.

While building the Netgram tool, we considered the following design principles:

- Each community is represented by a circle whose size is proportional to the number of nodes in that community at a given time-stamp *t*.
- The evolution of communities between 2 time-stamps is represented by a dashed line.
- Lines follow a straight path if there is no merge or split event during the entire course of evolution for a given community.
- If a part of a community say $C_{i}^{t}$ merges into another community say $C_{j}^{t+1}$ i.e. a merge event has occurred at time *t* + 1 then a dashed line is drawn from $C_{i}^{t}$ at time *t* to the community it merges at time *t* + 1.
- If a part of a community say $C_{i}^{t}$ splits into another community say $C_{j}^{t+1}$ i.e. a split event has occurred at time *t* then a dashed line is drawn from $C_{i}^{t}$ at time *t* to the community it merges at time *t* + 1.

We also take into account the following aesthetic conditions as suggested in [36] when designing a visualization tool:

- Minimize line cross-overs.
- Minimize screen space.

**Fig 6:**
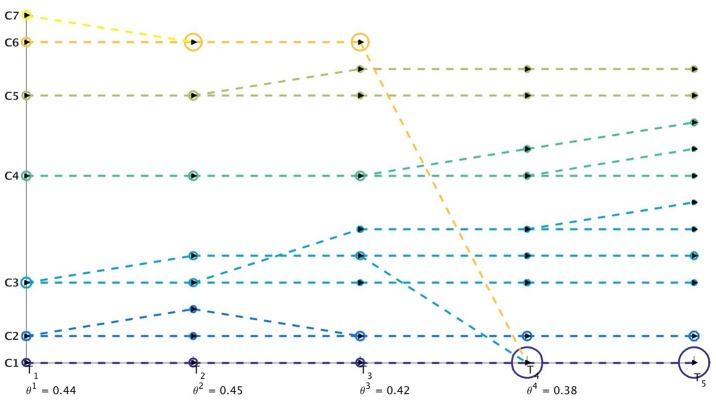
Visualization of evolution of communities obtained from OSLOM [11] method for Mergesplit dataset using Netgram. There are 11 cross-overs in this figure which is generated by passing the original order of partitions i.e 𝓞 = {1, 2, 3, 4, 5, 6, 7} to Algorithm 2.

**Fig 7:**
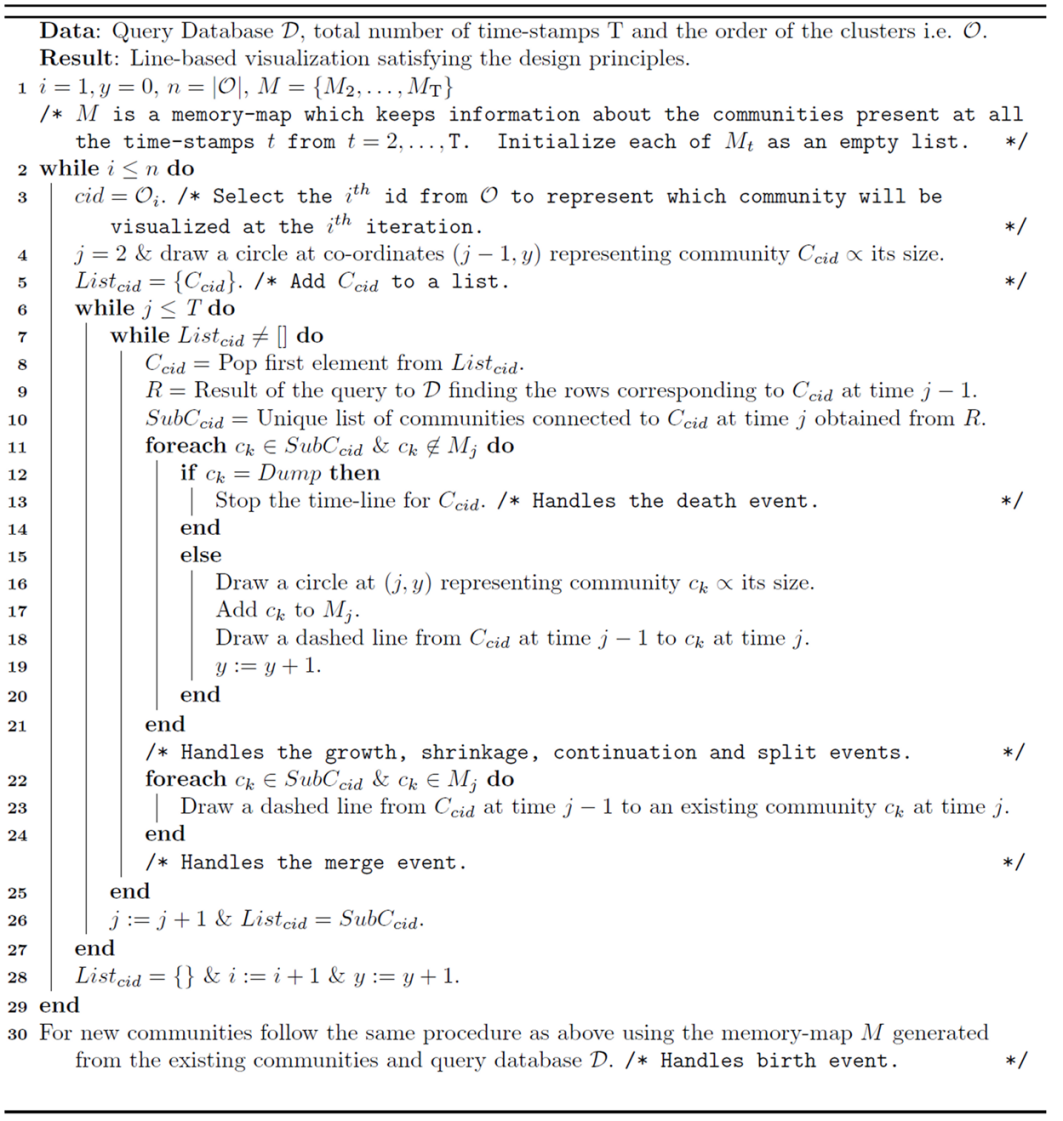
Algorithm 2: Netgram Visualization Layout.

These layout guidelines lead to a combinatorial problem with respect to the aesthetic conditions under the constraints of the design principles [36]. A similar problem for storyline visualization for streaming data was proposed in a constrained optimization framework in [36, 37]. In [36, 37] the authors try to visualize individuals (or groups) in a storyline. However, there is no event like merging or splitting of groups rather the groups come close together or move far from each other. Moreover, we can display additional information like size/density of communities at different time-stamps from the size of the circle representing those communities at these time-stamps. Fig 6 visualizes the evolution of communities over 5 time-stamps for the synthetic Mergesplit dataset using the community information obtained from OSLOM [11] method along with the original order of clusters in 𝓒^1^ by providing this information to Algorithm 2 (Fig 7).

The evolutionary clustering algorithm initially provides the community labels for all the nodes at a given time-stamp *t*. Using the query database 𝓓 and the initial order of the partitions in 𝓒^1^ i.e. 𝓞 = {1, 2, …, *n*} where *n* represents the total number of communities in the 1^*st*^ time-stamp, we propose a simple algorithm explained in Fig 7 that satisfies all the design principles and the aesthetic criteria except the minimization of line cross-overs. By using line based visualization instead of surface based visualization we minimize the screen space.

**Fig 8:**
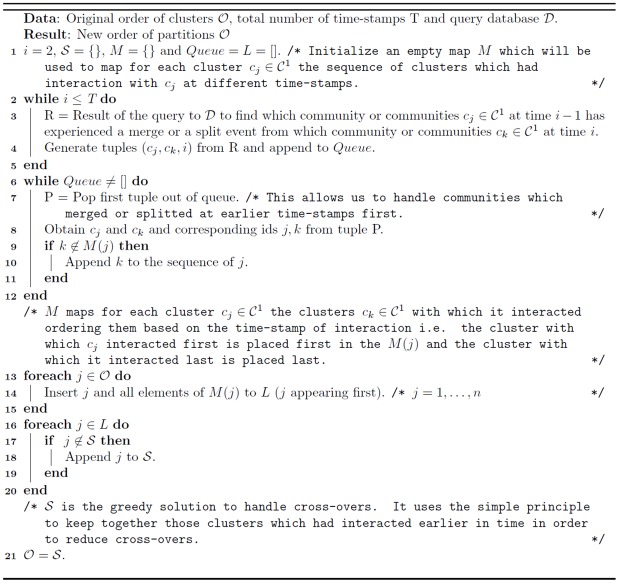
Algorithm 3: Greedy Solution to Handle Cross-Overs.

We now explain the concept of a line cross-over. A line cross-over generally occurs in case of a split or merge event. For example, in case of Fig 6, community **C6** merges into community **C1** at time-stamp T_4_. However, the line showing the merge event crosses over the time-line of communities **C5, C4, C3, C2** and its branches i.e. 8 lines in total. Similarly, community **C3** has one cross-over at time-stamp T_3_ during a split event and 2 cross-overs at time-stamp T_4_ due to a merge event with community **C1**. Thus, in total there are 11 cross-overs using the initial order of clusters i.e. 𝓞 = {1, 2, 3, 4, 5, 6, 7}. However, if we place **C6** next to cluster **C1** and community **C7** next to **C6** and maintain the order of the remaining communities i.e. 𝓞 = {1, 6, 7, 2, 3, 4, 5}, we can already reduce the number of line cross-overs to 3. We provide a greedy solution in Algorithm 3 (Fig 8) to sequence the order of clusters/partitions in 𝓒^1^ such that there are fewer line cross-overs in comparison to the initial order of the clusters i.e. 𝓞 = {1, …, *n*} provided by the evolutionary community detection technique.

**Fig 9:**
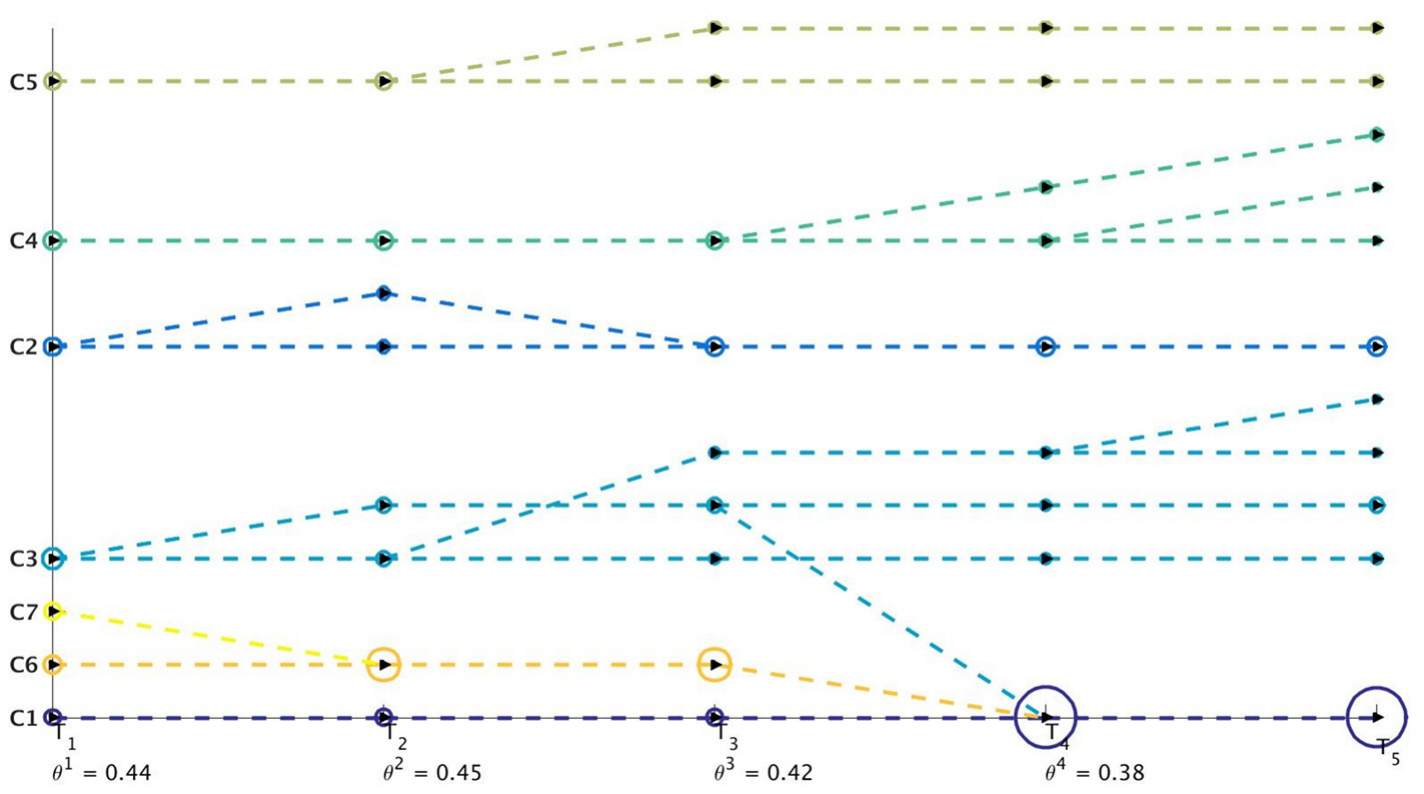
Visualization of evolution of communities for Mergesplit dataset using the refined order of partition 𝓞 obtained from Algorithm 3 and then passed to Algorithm 2. The revised ordering of partitions results in just 2 cross-overs as depicted in this figure.

**Fig 10:**
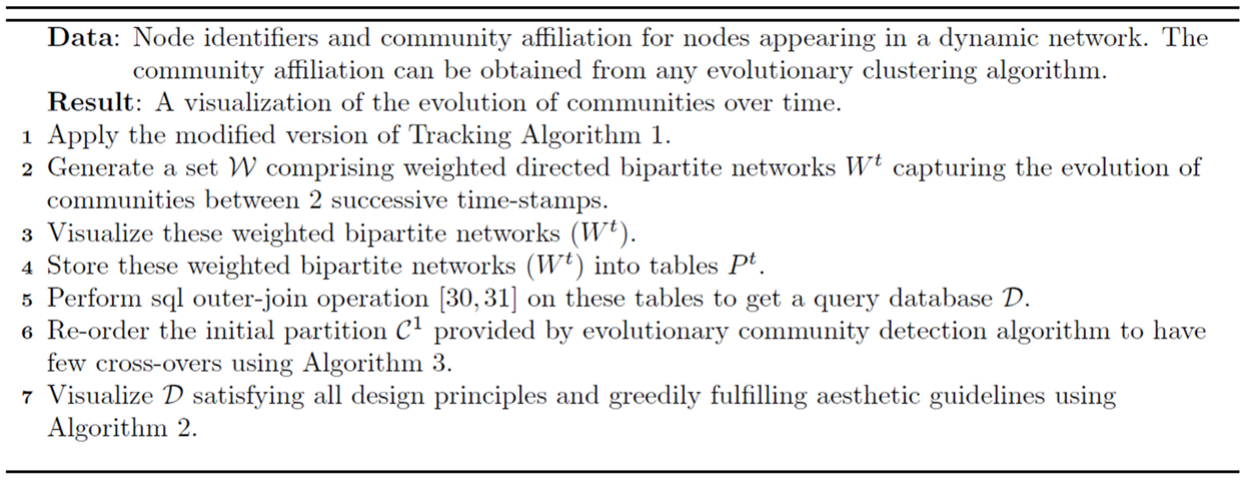
Steps undertaken by the Netgram tool for visualization.

Fig 9 illustrates the effect of applying Algorithm 3 on the initial order of partitions in 𝓒^1^ obtained from the OSLOM method [11] for the Mergesplit dataset and providing this new order 𝓞 to Algorithm 2 to perform visualization using Netgram. Fig 10 summarizes the steps undertaken by the Netgram tool for visualization of evolution of communities.

## Additional Provisions

We provide the user with some additional facilities. The user can provide two parameters *ρ* and *ν* as input. The former specifies the minimum weight for an edge in the bipartite network *W* ^*i*^ for all time-stamps *i* = 1, …, T-1. Netgram will then visualize only those edges whose weights are greater than or equal to *ρ*. This allows the user to interact with the Netgram tool and focus on certain communities which are less prone to random noisy events and have edges with weights above *ρ* during their entire evolution period.

The latter (*ν*) is a threshold which defines the tolerance level allowed for *ρ* to differ from average *θ* ^*i*^ for all the time-stamps *i* = 1, …, T-1. Average *θ* ^*i*^ is defined as $\mu_{\theta }=\frac{\sum_{i=1}^{\text{T-1}}\theta^{i}}{\text{T-1}}$. If ∣*ρ* − *μ*_*θ*_∣ ≤ *ν*, we set *θ* ^*i*^ = *ρ* for all time-stamps else we maintain the original value of *θ* ^*t*^ as obtained from Algorithm 1. This tolerance level prevents removal of too many edges from each *W* ^*i*^ and prevents generating a near empty visualization plot. In our experiments we set the tolerance level value *ν* ≤ 0.1 thus not allowing too much variation between user-specified *ρ* and *μ*_*θ*_ but at the same time allowing some non-essential edges to be removed from the visualization tool.

We also provide the user with an additional facility which allows to visualize the network configuration at a given time-stamp *t*. For each time-stamp *t* we plot the network using the community information and the edge flow between the communities. Each community is plotted as a circular disc and the size of this circular disc is proportional to the number of nodes within the community. These communities are connected to each other using edges. The weight of the edges is proportional to the total number of edges flowing from one community to another. These edge weights are normalized to take a values between [0, 1]. This is done by taking the ratio of the number of edges flowing between 2 communities to the maximum number of edges flowing between any 2 communities among the set of communities at this given time-stamp *t*. The edges are displayed as lines connecting 2 communities and are plotted in gray-scale format. Edges with weight close to 0 are represented with whiter shades whereas those closer to 1 as drawn with darker shades of gray. In each row of the plot we can showcase the network configuration for a maximum of 5 time intervals. Using this visualization technique, we can observe significant events like birth, death, growth, shrinkage, continuation and split. However, the visualization of a merge event is not feasible in this scheme.

**Fig 11:**
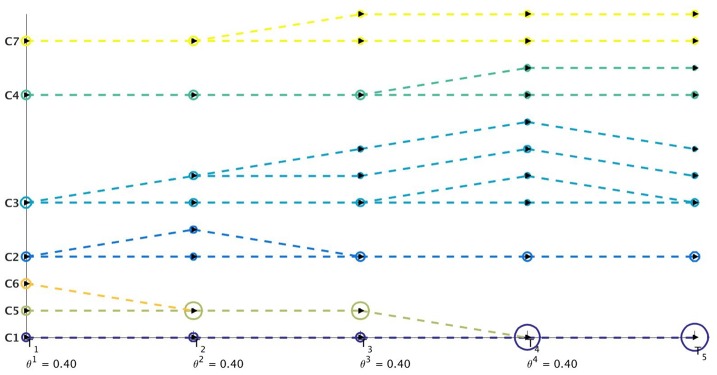
Visualization of communities for Mergesplit dataset obtained by Louvain [5] method keeping *ρ* = 0.4 and *ν* = 0.1. The value of *μ*_*θ*_ = 0.42 and since ∣*ρ* − *μ*_*θ*_∣ < *ν*, so we use *ρ* as the minimum weight of an edge for all time-stamps *t* = 1, …, T-1 as depicted in the figure. We observe that most of the edges are retained for this value of *ρ* as all edge weights are *w*(V^*t*^(*i*, *j*)) ≥ *ρ*.

**Fig 12:**
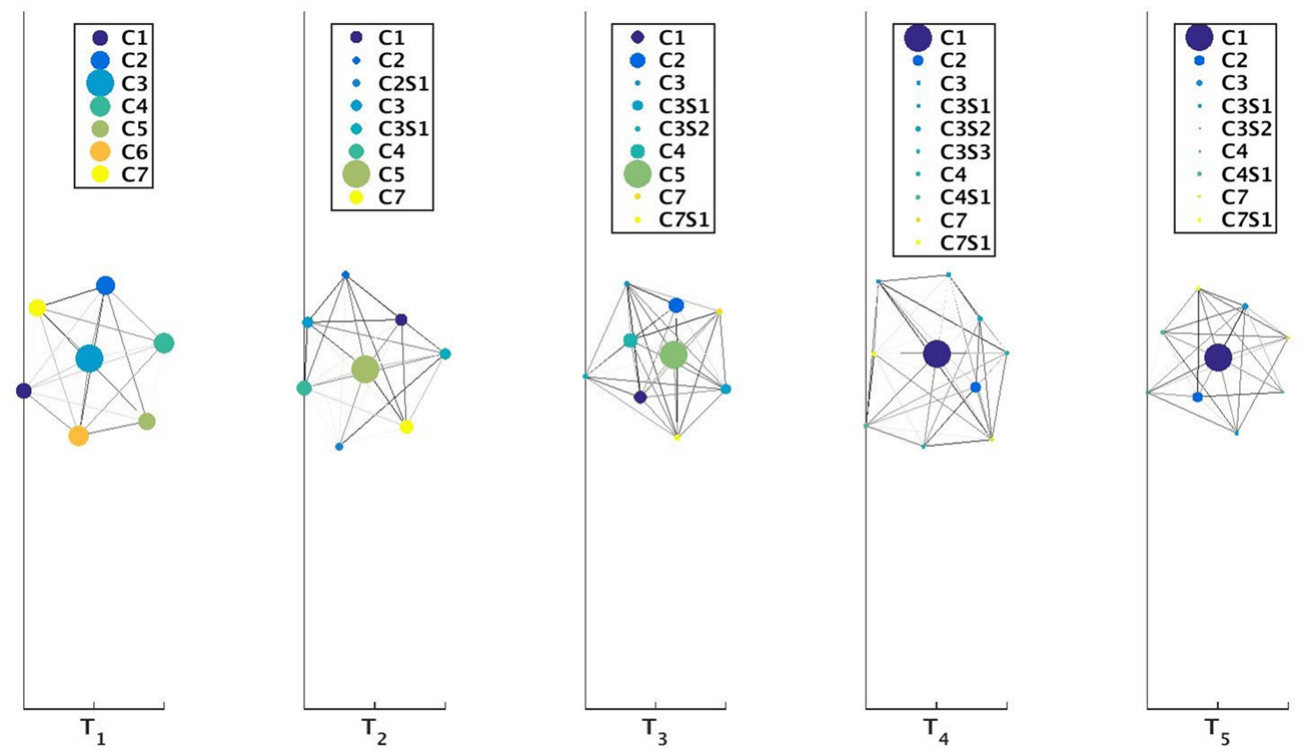
Visualization of network for Mergesplit dataset corresponding to Fig 11. The circular discs represent the communities with size proportional to number of nodes in these communities. From the legend of the subplot at time-stamp T_2_ we observe that community **C2** had experienced a split event at time-stamp T_1_ generating communities **C2** and **C2S1**. Here **CiSj** represents (*j* + 1)^*th*^ community appearing from **Ci**. Darker shaded edges represent communication between 2 clusters.

**Fig 13:**
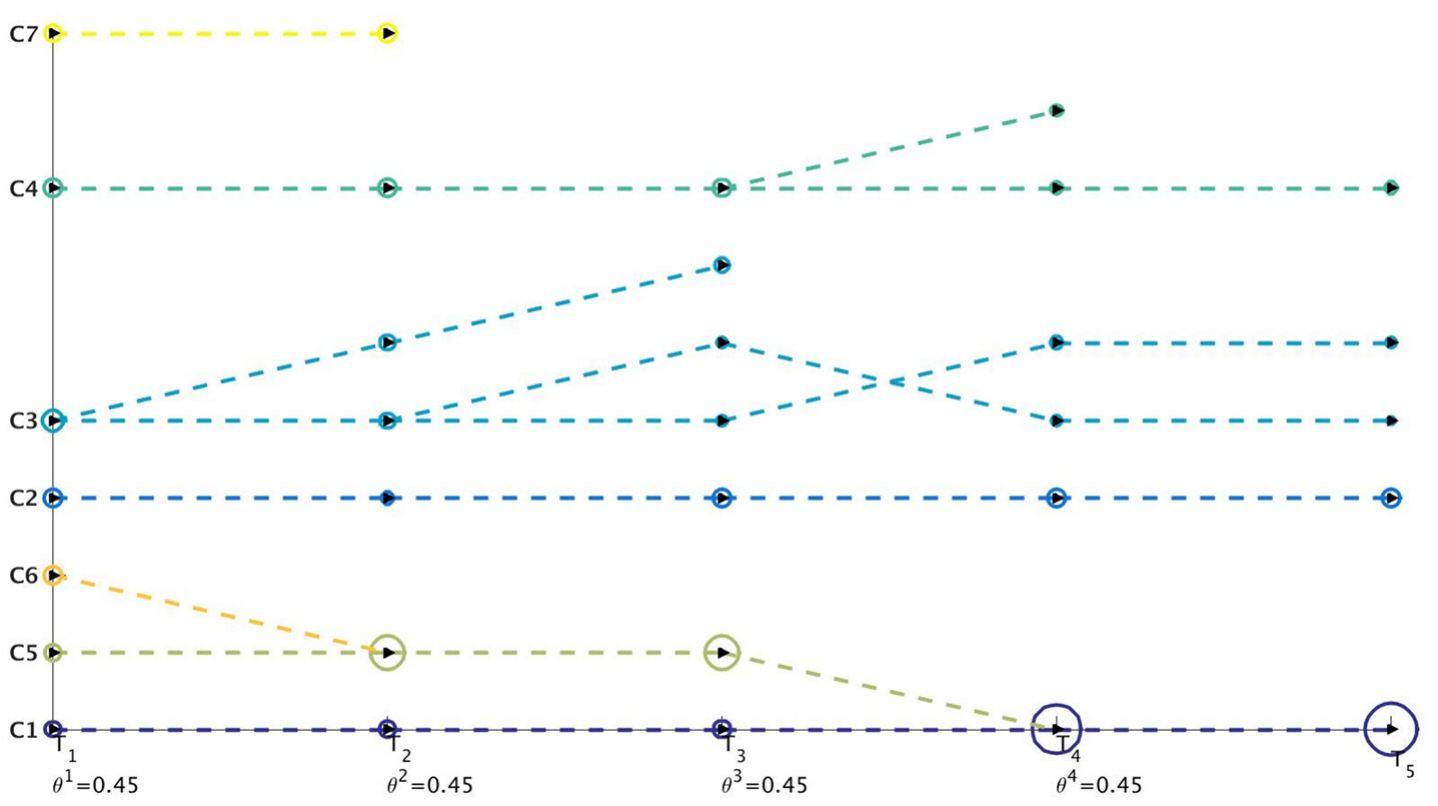
Visualization of communities for Mergesplit dataset obtained by Louvain [5] method keeping *ρ* = 0.45 and *ν* = 0.1. We observe that several edges have been removed in comparison to Fig 11. The life time of community **C7** has been shortened to just 2 time-stamps and some edges with weights less than *ρ* have been removed from community **C3**.

**Fig 14:**
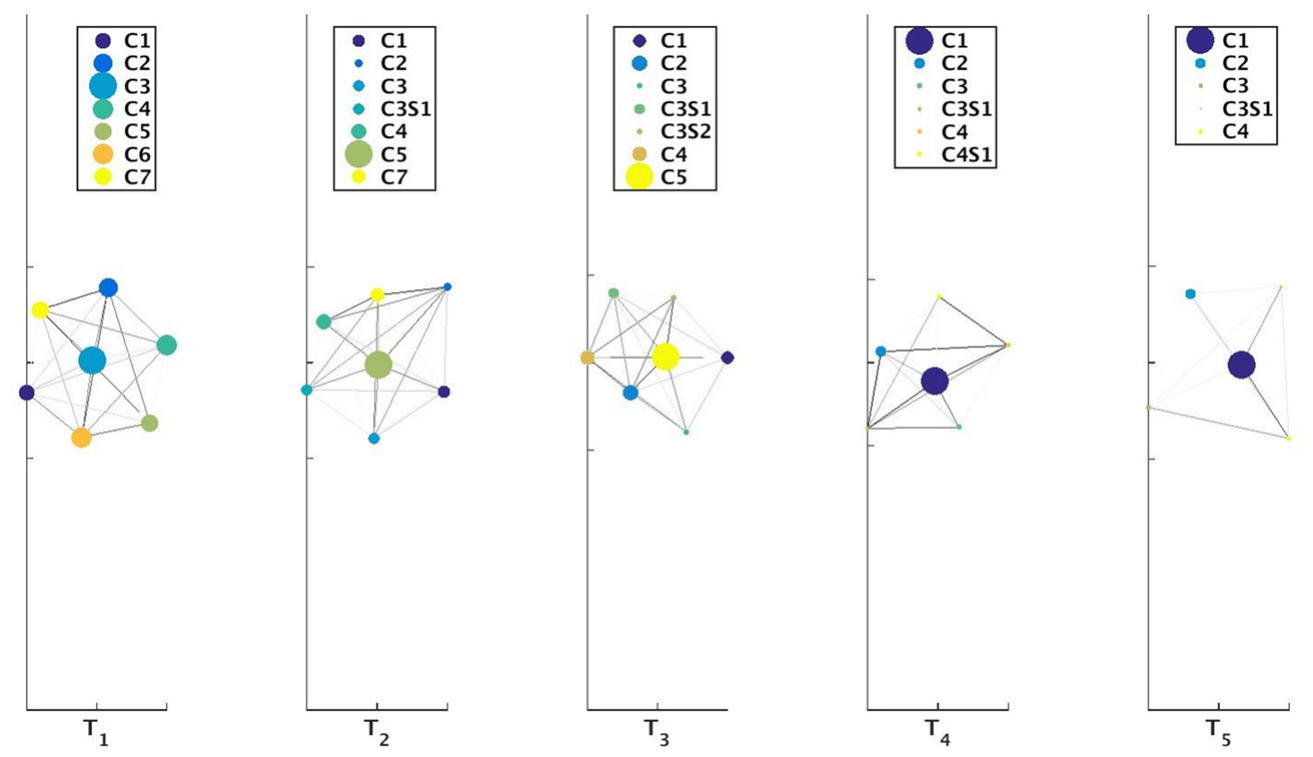
Visualization of network for Mergesplit dataset corresponding to Fig 13. The circular discs represent the communities with size proportional to number of nodes in these communities.

**Fig 15:**
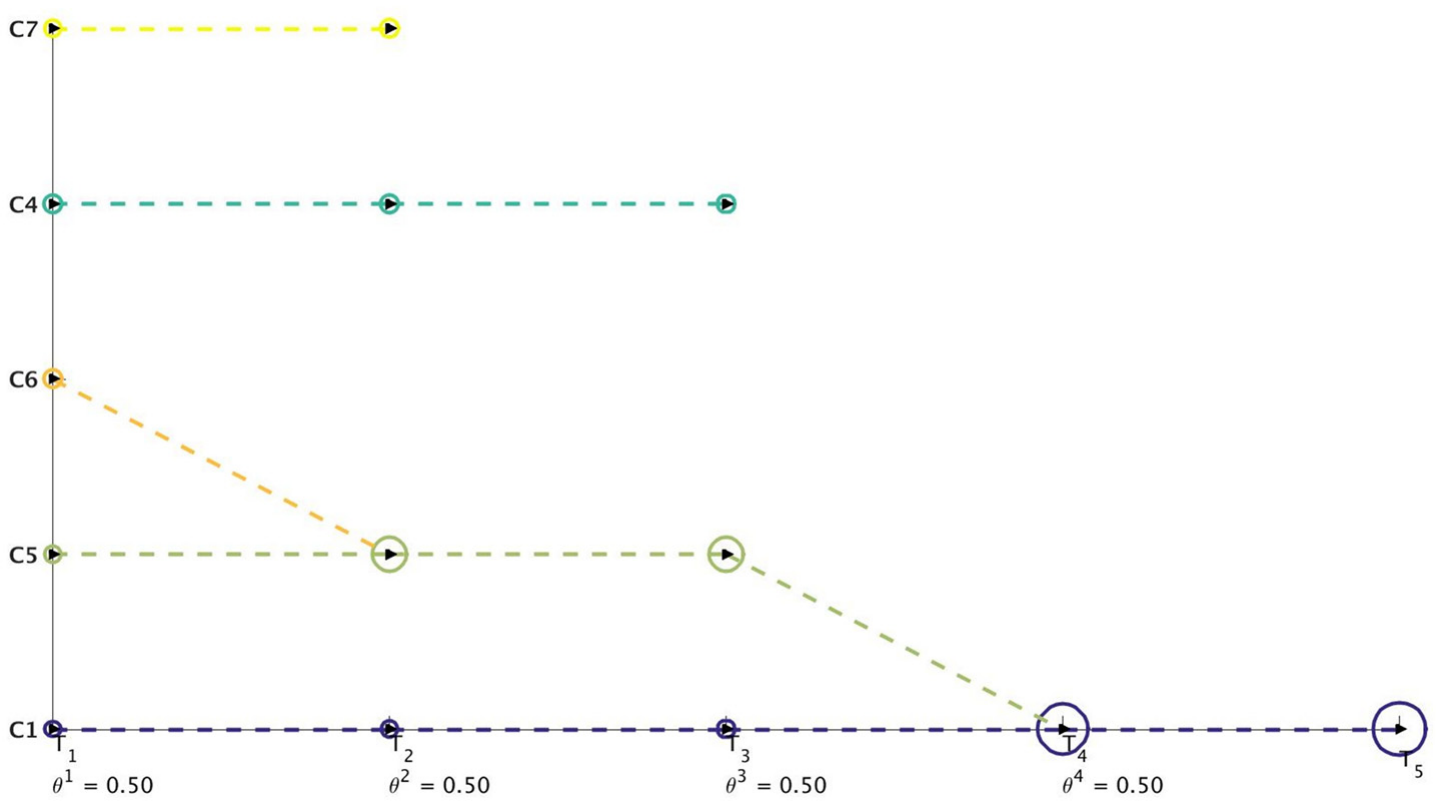
Visualization of communities for Mergesplit dataset obtained by Louvain [5] method keeping *ρ* = 0.5 and *ν* = 0.1. We observe that when *ρ* = 0.5 most of the edges are removed for Mergesplit dataset. Communities **C2** and **C3** no longer are part of the palette and life time of communities **C4** and **C7** are shortened. We can also observe that communities **C1**, **C5** and **C6** remain unchanged in comparison to Figs 11 and 13 indicating these communities have more stable evolution.

**Fig 16:**
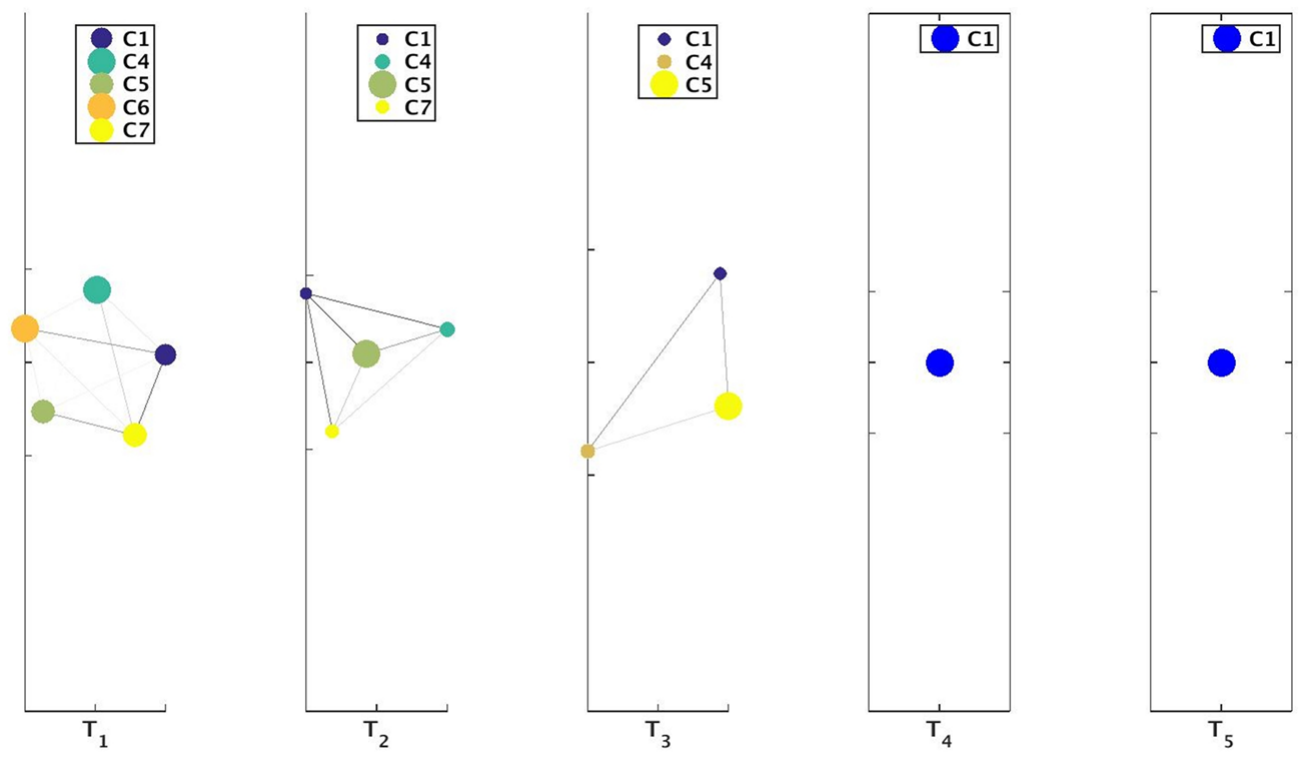
Visualization of network for Mergesplit dataset corresponding to Fig 15. The circular discs represent the communities with size proportional to number of nodes in these communities. There exists only community **C1** for time-stamp T_4_ and T_5_ using *ρ* = 0.5. If only one community is present then it is visualized using a blue-colored disc.

We illustrate the usage of these parameters *ρ* and *ν* for 3 settings in case of Mergesplit dataset using the community information obtained from Louvain method. We keep the *ν* value fixed at 0.1 and set *ρ* ∈ {0.4, 0.45, 0.5} to obtain results as depicted in Figs 11, 12, 13, 14, 15 and 16 respectively.

## Experiments

We first provide a brief description of the datasets used in this paper. In the previous sections, we have seen the use of several synthetic datasets including:

- **Birthdeath**—A dataset comprising 5 dynamic networks. There are 13 communities in the network at time-stamp *t*_1_. At every time-stamp *t*_*i*_, *i* > 2 there is death of one community and at each time-stamp *t*_*i*_ > 3 there is birth of one community. The number of nodes in the networks decrease from 1,000 to 886 over time. We illustrate the evolution of communities for this dataset using the MKSC algorithm [28, 29] in Fig 2.
- **Hide**—A dataset comprised of 5 dynamic networks. There are 7 communities in the network at time *t*_1_. At each time-stamp *t*_*i*_, one community disappears as was shown in the visualization of the weighted bipartite networks in Fig 5.
- **Mergesplit**—A dataset comprising 5 dynamic networks. There are 7 communities in the network at time *t*_1_ and 1,000 nodes in each of the 5 snapshots of this dataset. At each time-stamp *t*_*i*_, there is 1 merge and 2 split events. A visualization of the evolution of the communities obtained by OSLOM [11] method for this dataset was provided in Fig 6.

These networks were generated using the software available at https://github.com/derekgreene/dynamic-community. We also experimented on real-life datasets and a synthetic large scale dataset (to show the scalability of the Netgram tool) which are described below:

- **Reality**—This dataset monitors the cellphone activity of people from 2 different labs in MIT [38]. This dataset consists of networks where the node represents a user whose cellphone periodically scans for other cellphones via Bluetooth. The weight of the edge between two nodes is equal to the number of intervals for which the 2 users are in close proximity to each other. Each snapshot corresponds to a weighted network which records the activities of the users over a period of 1 week. There is a total 32 meaningful snapshots and total number of users monitored over this time-span is 94. However, not all users are present in each weighted network. The smallest network comprised of 21 people and largest network had 88 people.
- **NIPS**—This dataset consists of information about 1,500 papers published in the Advances in Neural Information Processing Systems (NIPS) conference starting from 1987 to 1998. The dataset is part of the Bag of Words Dataset [39] from the UCI repository http://archive.ics.uci.edu/ml/. From this dataset, we first separate out the papers published in each year starting from 1987 till 1998. We then create a TF-IDF model [40] using which we remove out irrelevant words from the documents. We then create a word-word graph for each time-stamp where the weight of the edges in the network is proportional to occurrence of 2 words together in all the documents for that year.
- **Weather**—The website http://www.wunderground.com/ was utilized to obtain weather information for about 9 months for 23 European cities. The time period over which this data spanned was from January 2012 to October 2012. For each city for each day we collected information about 20 attributes. The goal was to cluster these cities using weekly information about these cities i.e. one snapshot consists of 23 cities and 140 variables where 20 variables from each day are concatenated together. Thus in total we have 40 snapshots corresponding to 40 weeks.
- **Big**—This dataset consists of 5 networks where the network at the first time-stamp has 1 million nodes. There are 10 communities in the network at time *t*_1_. The number of nodes decrease over time. The dataset exhibits several significant events like merge, split and death of communities. It was also generated from the software provided in [22].

The Netgram toolkit is most suitable for visualization of a small number of large sized clusters. This is because in case of a large number of clusters the palette for plotting will become too cluttered and it becomes difficult to keep track of the evolution of individual communities. Thus, in case of a hierarchical evolutionary clustering algorithm like OSLOM [11] or Louvain [5] method, Netgram is most suitable for visualization of giant-connected components at coarser levels of hierarchy.

**Fig 17:**
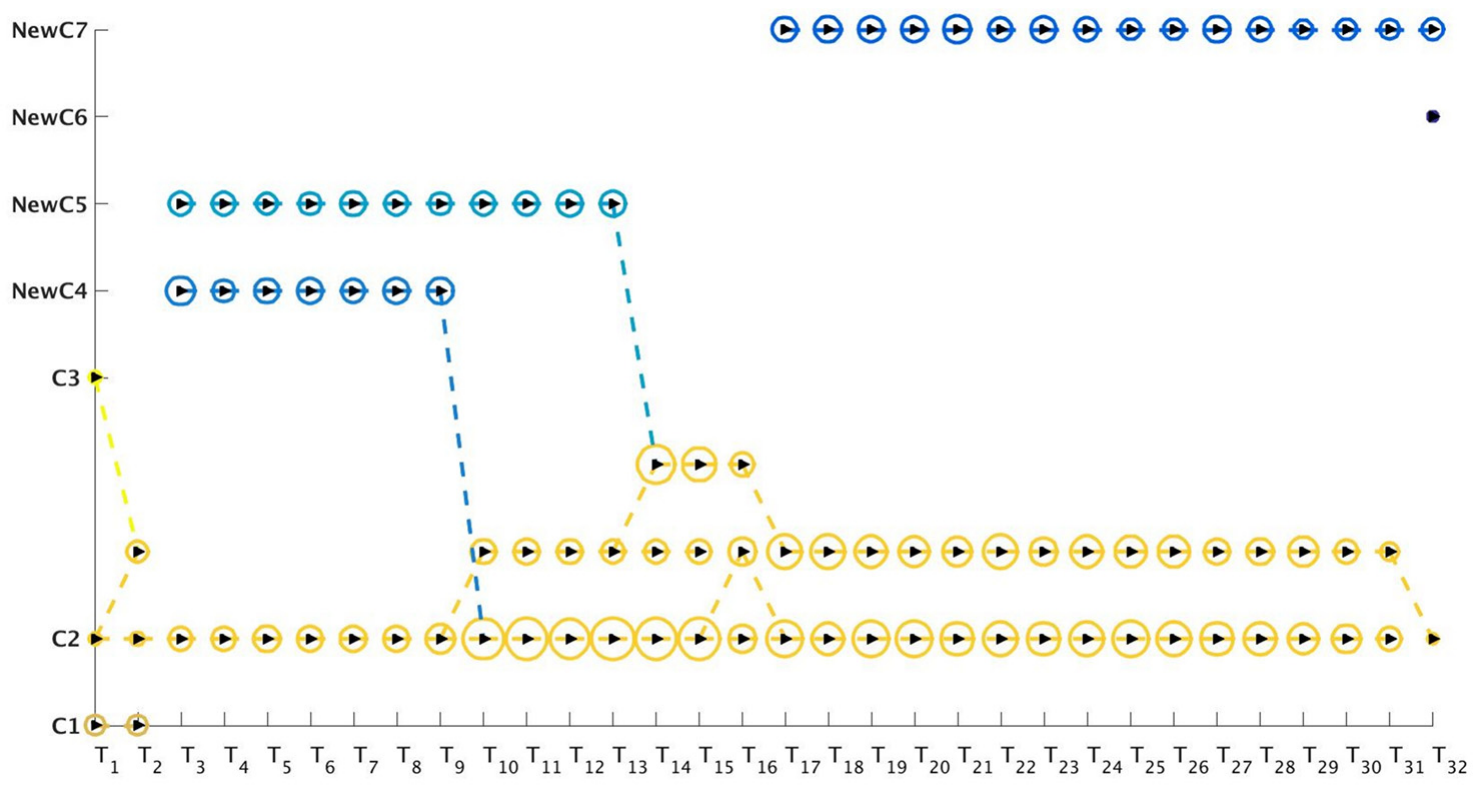
Visualization of evolution of communities generated by Evolutionary *k*-means [23] method using the Netgram tool for the Reality dataset. We mostly observe continuation of communities. However, communities **C1** and **C3** both disappear at time-stamp T_2_ and new communities **NewC4** and **NewC5** appear at time-stamp T_3_. By time-stamp T_14_ all communities merge into branches of community **C2**. New communities **NewC6** and **NewC7** appear at time-stamps T_18_ & T_32_ respectively.

**Fig 18:**
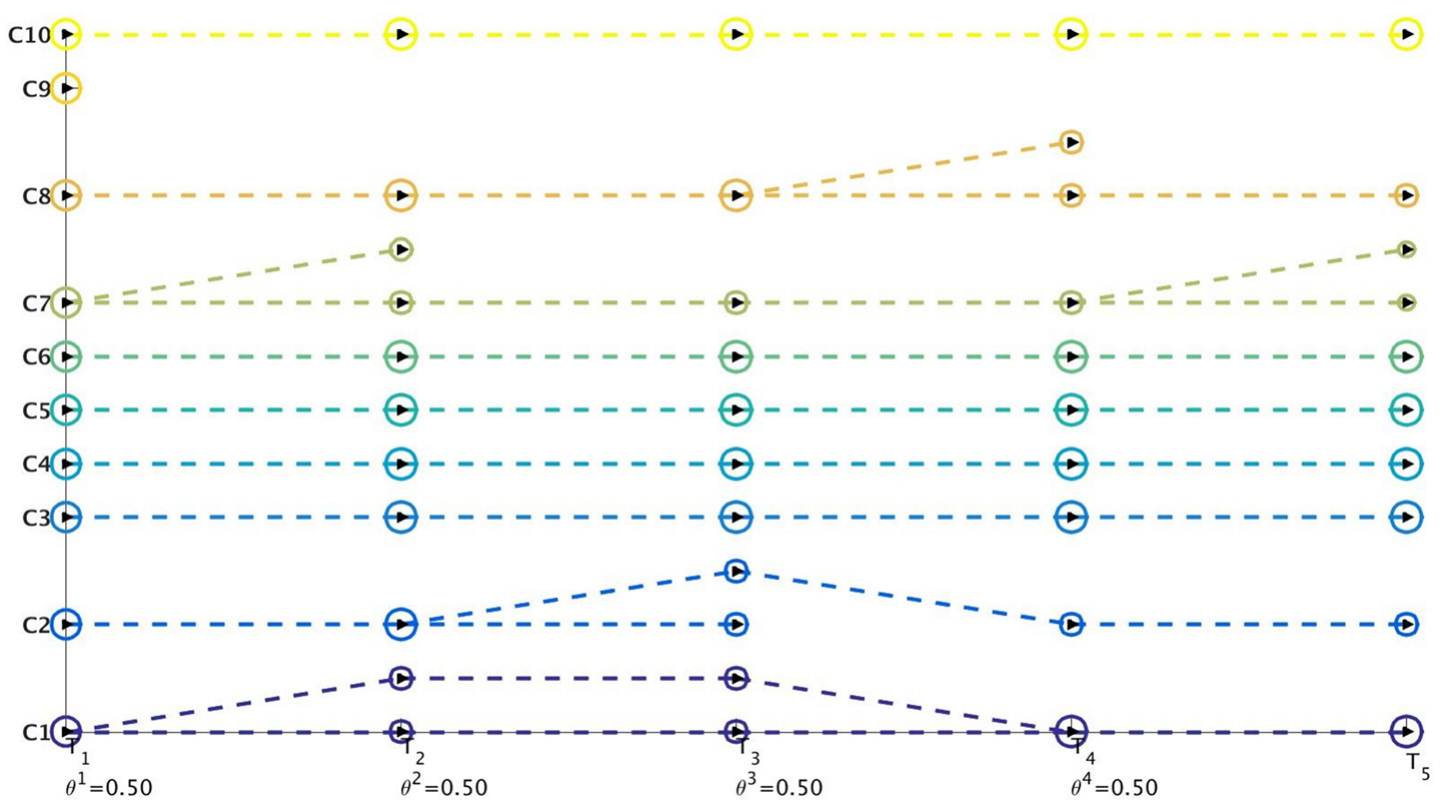
Visualization of evolution of communities generated by the Louvain [5] method using the Netgram tool for the Big dataset. We can observe significant events like merge, split, death and shrinkage of communities. For example, we can observe the split of communities **C7** and **C8** at time-stamp T_2_ and T_3_ respectively. Similarly, we can observe a merge event for cluster **C1** at time T_4_. We observe a general pattern of shrinkage for most of the communities in this dataset.

Figs 17 and 18 depict the evolution of communities for the Reality and the Big dataset respectively. In case of the Reality dataset, we use the Evolutionary *k*-Means technique introduced in [23] to obtain the community affiliation for all the nodes at different time-stamps. In case of the Big dataset, we use the Louvain method [5] to generate the community memberships for all the nodes in the network over different periods of time. We use the Louvain method as it can easily scale to 1 million nodes for community detection which is otherwise a difficult task for evolutionary clustering algorithms.

### NIPS Results

**Fig 19:**
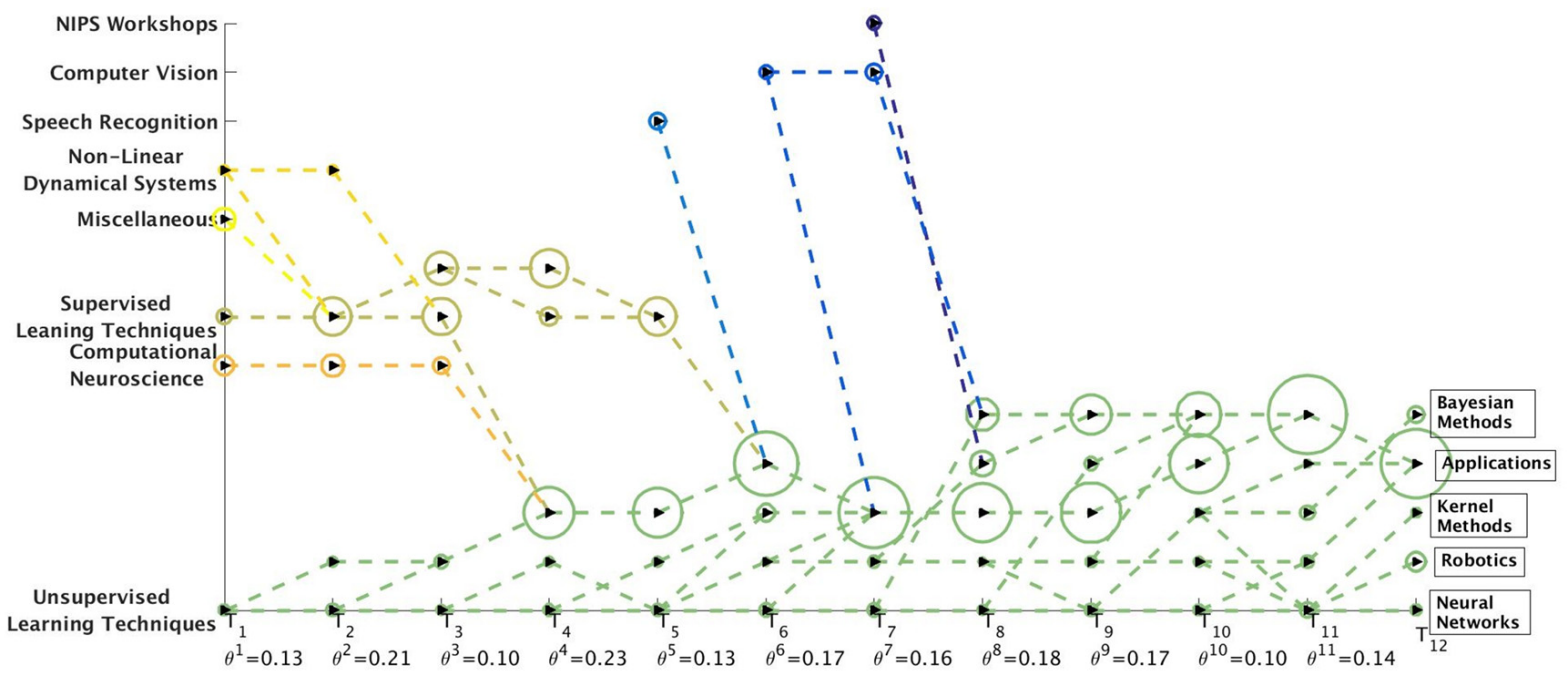
Visualization of evolution of communities for the NIPS dataset by Netgram toolkit.

We obtain the communities from the word-word graph for the NIPS dataset using the MKSC [28] algorithm. The MKSC technique identified 5 communities at each time-stamp for this dataset. However, as we observe from Fig 19, the birth of several new communities was detected by the MKSC algorithm. We observe the appearance of disciplines like Speech Recognition and Computer Vision as distinguishable and distinct communities (in comparison to Supervised Learning Techniques) in 1991 and 1992 respectively. Fig 19 illustrates that the Netgram tool also detected the inception of NIPS Workshops in the year 1993. By the year 1998, we have specialized disciplines like Neural Networks, Robotics, Kernel Methods and Bayesian Methods which are combinations of Supervised Learning Techniques and Unsupervised Learning Techniques as observed from Fig 19.

**Fig 20:**
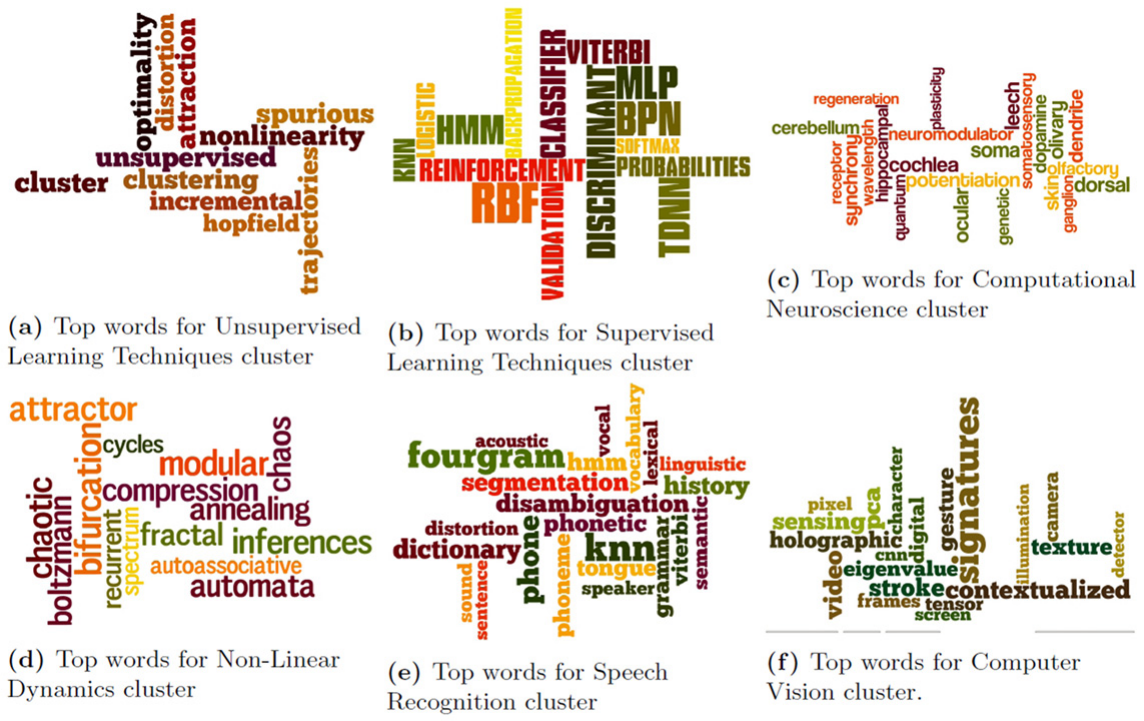
Word clouds for top words in the communities detected by MKSC method [28, 29] over the first few time-stamps for the NIPS dataset.

**Fig 21:**
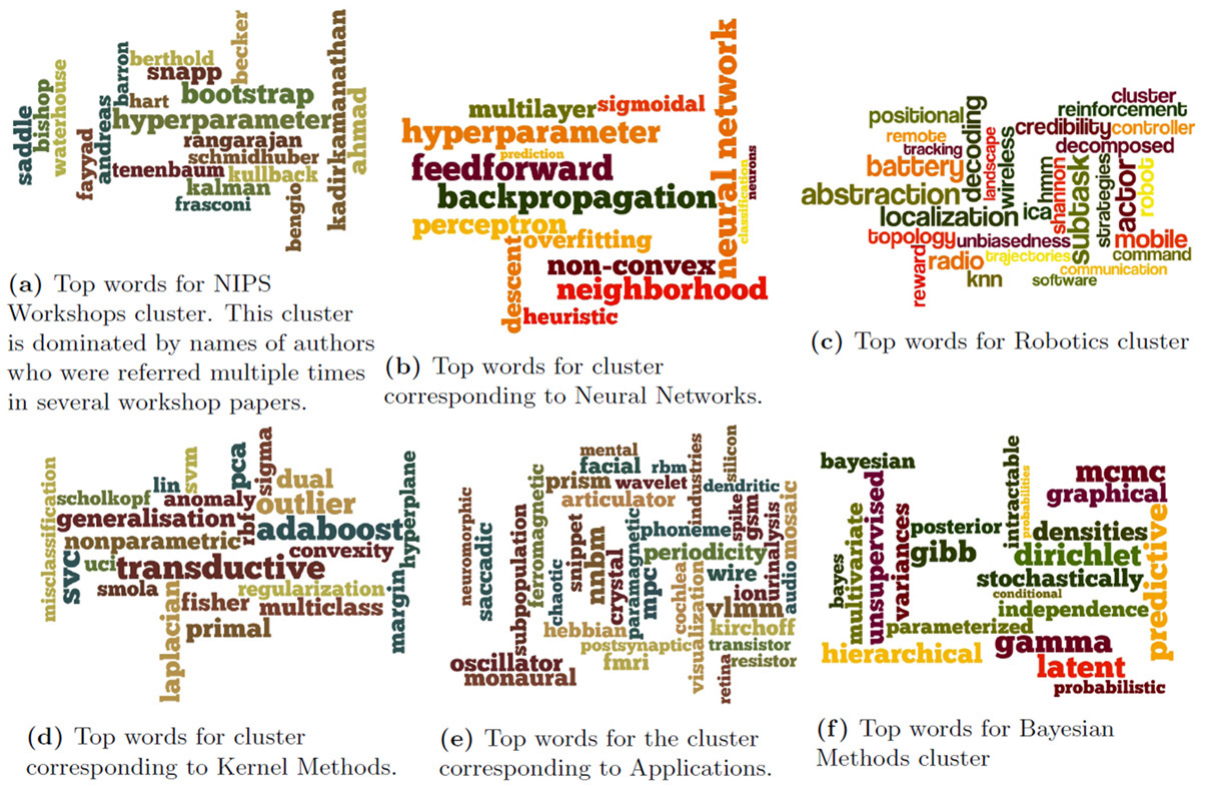
Word clouds for top words in the communities detected by MKSC method [28, 29] over the last few time-stamps for the NIPS dataset.

Since we use a TF-IDF model [40] to obtain the word-word graph on which we perform the community detection for the NIPS dataset, we can identify the top words (based on TF-IDF [40]) in these communities. In Figs 20 and 21, we showcase these top words corresponding to the communities detected by MKSC algorithm [28, 29].

### Weather

**Fig 22:**
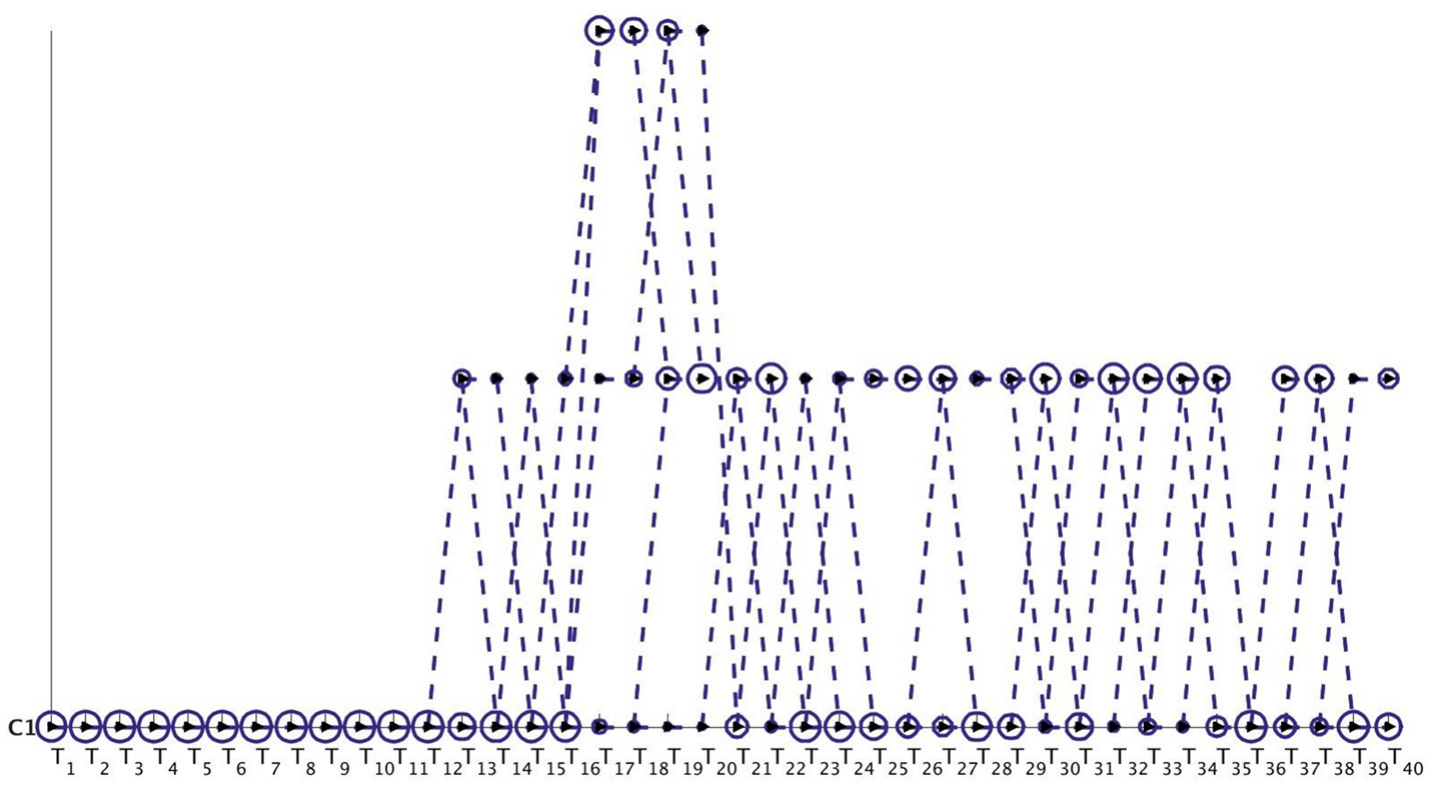
Visualization of evolution of clusters obtained using MKSC [28] algorithm for the weather dataset by Netgram toolkit. The y-axis showcases that all the 23 European cities belong to one cluster **C1** at time T_1_. The cluster **C1** splits into 2 or more clusters at later time-stamps. But, since all these splitted clusters originate from **C1** and the set of cities (nodes) remain constant, they are plotted with the same colour as **C1**. We provided *ρ* = 0.5 and *ν* = 0.1 as parameters to visualize only significant events and for all time-stamps we observe that ∣*ρ* − *μ*_*θ*_∣ ≤ *ν*. Thus, *θ* ^*t*^ = *ρ* = 0.5 for all time-stamps *t* < 40 and is kept constant. So, we don’t specify the constant threshold value on the x-axis and prevent the visualization palette from getting over-crowded.

For the weather dataset for each day we gathered information about attributes like maximum, minimum and mean temperature, dew point, humidity, sea level pressure, visibility, wind speed respectively and precipitation and wind direction for each city. The 23 European cities for which the data was gathered included Amsterdam, Antwerpen, Athens, Berlin, Brussels, Dortmund, Dublin, Eindhoven, Frankfurt, Groningen, Hamburg, Liege, Lisbon, London, Madrid, Milan, Nantes, Paris, Prague, Rome, Toulouse, Vienna and Zurich. We run the MKSC [28, 29] algorithm to obtain the communities for different time-stamps. The MKSC technique takes into account temporal smoothness [19] using the memory. Fig 22 depicts the result obtained for the weather dataset using MKSC algorithm.

Initially community **C1** consists of all the 23 European cities. However, after beginning of April (T_13_) cluster **C1** splits into 2 or more prominent communities for all later time-stamps. This suggests that winter pattern in most of the European cities are nearly same. During the first week of April (T_13_) the smaller cluster comprises cities like Milan, Rome, Nantes, Paris, Prague, Toulouse and Vienna which indicated that outbreak of spring in these cities are similar to each other and different from other European cities. During mid May (T_19_), we observe 3 communities where one cluster was just the city of Dublin, the other 2 clusters consisted of Amsterdam, Antwerpen, Brussels, Berlin, Dortmund, Eindhoven, Frankfurt, Groningen, Hamburg, Liege, London, Nantes, Paris and Athens, Lisbon, Madrid, Milan, Prague, Rome, Toulouse, Vienna, Zurich respectively. This clustering information clearly distinguishes the weather pattern of western European cities from cities of southern Europe (except Prague).

The Netgram tool is available for usage at http://www.esat.kuleuven.be/stadius/ADB/mall/downloads/Netgram_Tool.zip.

## Conclusion

In this paper we proposed a visualization toolkit Netgram which can be used to depict the evolution of communities in dynamic networks over time. Netgram was used to illustrate the occurrence of significant events like birth, death, merge, split, expansion, shrinkage and continuation of communities over time. Netgram can be used as a post-processing step to any evolutionary clustering algorithm. Netgram provides a simple line-based visualization tool for tracking the evolution of communities for various datasets. The tracking of the evolution of communities was performed in such a way that there were only a few line cross-overs and efficient screen space usage (since we used dashed lines). We proposed a greedy solution to have fewer line cross-overs in comparison to the plot obtained by using the original order or partitions (𝓞) as provided by the evolutionary clustering algorithm.
